# Supplementary material for: Redox-active electrolyte-based printed ionologic devices
Source: Nat Commun. 2025 May 21;16:4725. doi: 10.1038/s41467-025-59746-7 (PMC12095779; doi:10.1038/s41467-025-59746-7)
Supplement: Supplementary file 1 — Supplementary Information [file 41467_2025_59746_MOESM1_ESM.pdf]

Supporting Information

Redox-active electrolyte based printed ionologic devices

Hanfeng Zhou[a], Przemyslaw Galek[a], Tianle Zheng[b], Panlong Li[a], Xiongjun Zhou[c], Congcong Liu[d], Jonas Kunigkeit[e], Katherina Haase[f], Yuxi Li[a], Jiang Qu[d], Ahmed Bahrawy[a], Peixun Xiong[a], Julia Grothe[a], Daria Mikhailova[d], Stefan CB Mannsfeld[f], Eike Brunner[e], and Stefan Kaskel[a,g*]*

[a]Inorganic Chemistry I, Technische Universität Dresden, Bergstrasse 66, 01069 Dresden (Germany)

[b]Department of Chemistry, Shanghai University, Guangyan Rd 123, 200444 Shanghai (China)

[c]Mechanical and Electrical Engineering, Kunming University of Science and Technology, Jingming South 727, 650093 Kunming (China)

[d]Leibniz Institute for Solid State and Materials Research, Helmholtzstraße 20, 01069 Dresden (Germany)

[e]Bioanalytical Chemistry, Technische Universität Dresden, Bergstrasse 66, 01069 Dresden (Germany)

[f]Center for Advancing Electronics Dresden, Technische Universität Dresden, Helmholtzstraße 18, 01062 Dresden (Germany)

[g]Fraunhofer Institute for Material and Beam Technology, Winterbergstraße 28, 01277 Dresden (Germany)

Table of contents

1. Materials....................3
2. Electrochemical and related *in-situ* measurement....................4
3. Assembling the system....................5
4. Characterization of carbon materials....................6
5. The characterization of titanium mesh....................7
6. System configurations....................8
7. Density functional theory (DFT) calculations....................9
8. Concentration impact....................12
9. Details of charge storage mechanism....................13
10. Rectification ratios....................17
11. Symmetric systems....................19
12. MAS NMR spectroscopy....................20
13. The role of electrolyte....................22
14. Rectification mechanism....................25
15. Logic gates....................31
16. Comparing Systems....................42

1. Materials

Kynol Europe GmbH® (Germany) supplied the active carbon fibers ACC-5092-10 ($C_{0.70}$), which are based on a material called Novoloid. Pitch-based active carbon fiber A20 ($C_{1.5}$) was supplied by AD'ALL Co® (Japan). Carbon Molecular Sieve (CMS; $C_{0.55}$) was purchased from Guangdong New Energy Technology Co., Ltd.® (China). By convention, the peak pore size of different types of carbon is denoted by the subscript *x* in the chemical formula for that carbon (C_x). All chemicals were utilized exactly as they came into the chemical shipment without further purification. Carbon black AB208928 (50% compressed; 99,9%) was purchased from ABCR GmbH & Co® (Germany). Polytetrafluoroethylene (PTFE; average Mw ~534,000 by GPC), Titanium powder (Ti powder; 99.98%; Ti is used in a dispersed form to enable the printing process, allowing for the creation of a flexible and uniform Ti electrode surface), and phosphotungstic acid hydrate ($H_3[PW_{12}O_{40}]\cdot xH_2O$; product number: P4006) were purchased from Sigma Aldrich® (USA). Glassfiber separators GF/D were purchased from Whatman® (UK). Carbon black Super p MA-EN-CO-01 and titanium mesh (>99.5% of Ti; as a rigid solid electrode) were purchased from Canrd® (China). For titanium mesh, surface oxidation leads to a thin TiO_2 layer at the surface, and titanium dioxide (TiO_2) is positively electrostatically charged in an acidic electrolyte (pH < 6).[1,2]

2. Electrochemical and related *in-situ* measurement

Potentiostat/galvanostat VMP300 by Biologic® (France) was utilized to analyze the electrochemical CAPode behavior. Cyclic voltammetry (CV), galvanostatic cycling with potential limitation (GCPL), and chronoamperometry (CA) were used as techniques.

In-situ measurement: DXR SmartRaman Spectrometer (532 nm laser) was applied to collect Raman spectra. *In-situ* cylindrical quartz glass tube cell was prepared by inserting one WE (Ti mesh with 0.5 cm ×2.0 cm) and one CE (10 ± 2 mg carbon coated on Ti mesh with 0.5 cm × 2.0 cm, both separated by a PVDF separator). After assembly, the cell was filled with ≈300 μL of the respective electrolyte (1.5 cm). IVIUM pocketSTAT 2 potentiostat was connected to the in-situ tube cell for electrochemical testing. The different potentials (0, -0.4, -0.5, -0.6, -0.7, -0.8, -0.9, and -1.0 V) were applied to the cell and each potential was held for 600 s. Raman spectra were collected in real-time during potential cycling, with each spectrum registered for 120 s.

3. Assembling the system

The carbon electrodes were prepared by mixing 85 wt% porous carbon, 10 wt% carbon black AB208928 as a conductive agent and 5 wt% PTFE as a binder to yield a free-standing film. From this film, discs with a diameter of 10 mm were punched out. The thickness of the super-microporous carbon is 80 µm and for microporous (>1 nm) carbon is 150 µm. These two layers are separated by a glassfiber separator GF/D, and they are mounted on a carbon-coated Ti current collector (12 mm in diameter).

4. Characterization of carbon materials

Nitrogen physisorption was measured volumetrically at 77 K on a BELSORP apparatus (Microtrac MRB®). The AUTOSORB-iQ-C-XR from Quantachrome® was used to perform a physisorption study with argon at 87 K. The specific surface area (SSA) was determined using the Brunauer-Emmett-Teller (BET) method and the total pore volume was calculated using the cumulative results according to the density functional theory (DFT) for the partial pressure range of $0.05 < P/P_0 < 0.20$. The total pore volume up to 2 nm was used in DFT calculations to derive micropore volumes. The samples were degassed at 150°C for at least 12 h prior to measurement.

Fig. S1 presents physisorption isotherms for different tested activated carbons.

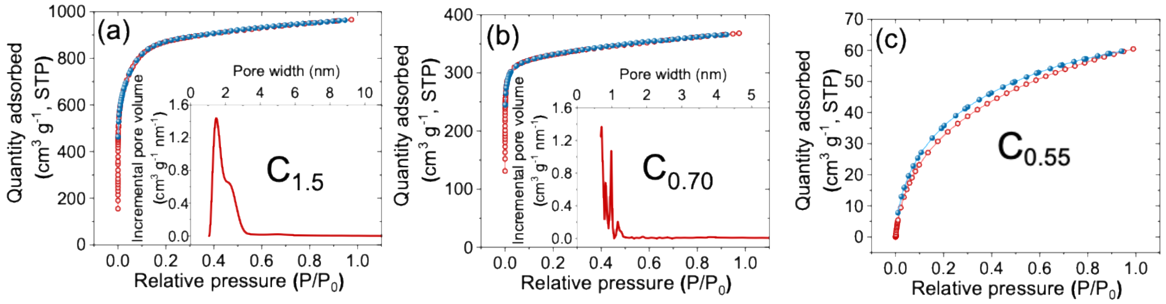

Fig. S1. N_2 physisorption isotherms at 77 K for (**a**) $C_{1.5}$ and (**b**) $C_{0.70}$ (pore size distribution on insets). (**c**) CO_2 physisorption isotherm at 260 K for $C_{0.55}$.

Tab. S1. Textural properties of the carbon materials employed.

Sample [a]	Symbol	S_A[b] ($m^2\ g^{-1}$)	V_{micro}[c] ($cm^3\ g^{-1}$)	V_{meso}[d] ($cm^3\ g^{-1}$)	V_{total}[e] ($cm^3\ g^{-1}$)	d_{peak} (nm)	$V_{>1.5\ nm}$ [f] ($cm^3\ g^{-1}$)
A20	$C_{1.5}$	2492	1.1	0.24	1.34	1.5	0.63
ACC-9052-10	$C_{0.70}$	1616	0.49	0.06	0.54	0.7	0.07

[a] Label C_x specifies x as the peak pore size (d_{peak}) from DFT theory. [b] SSA was calculated using BET method applied in the relative pressure range of 0.05 – 0.2. [c] V_{micro} is cumulative pore volume up to 2 nm. [d] V_{meso} is the difference between V_{total} and V_{micro}. [e] V_{total} is the cumulative pore volume. [f] $V_{>1.5\ nm}$ is the cumulative pore volume larger to 1.5 nm.

5. The characterization of titanium mesh

Ex-situ Raman spectroscopy was carried out at room temperature, and the signals were recorded by a LabRAM HR Evolution (Horiba) using a laser with an excitation wavelength of 458 nm. A long working distance (10.6 mm) with a 50-zoom objective was used, and the spot size of the laser beam was focused at 0.77 μm of Ti mesh. There is one remarkable peak for the Ti-O bond (310 cm^{-1}), which also confirms the existence of TiO_2 on the surface of the Ti mesh.[3]

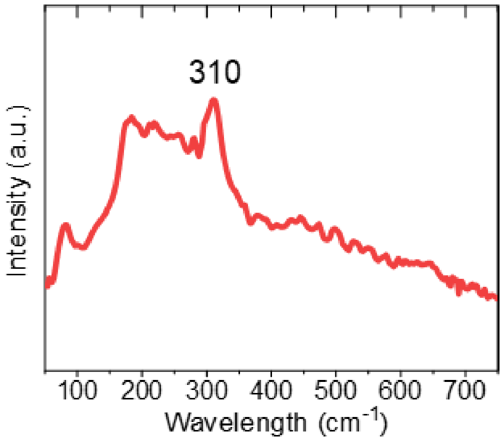

Fig. S2. The Raman spectrum of Ti mesh.

6. System configurations

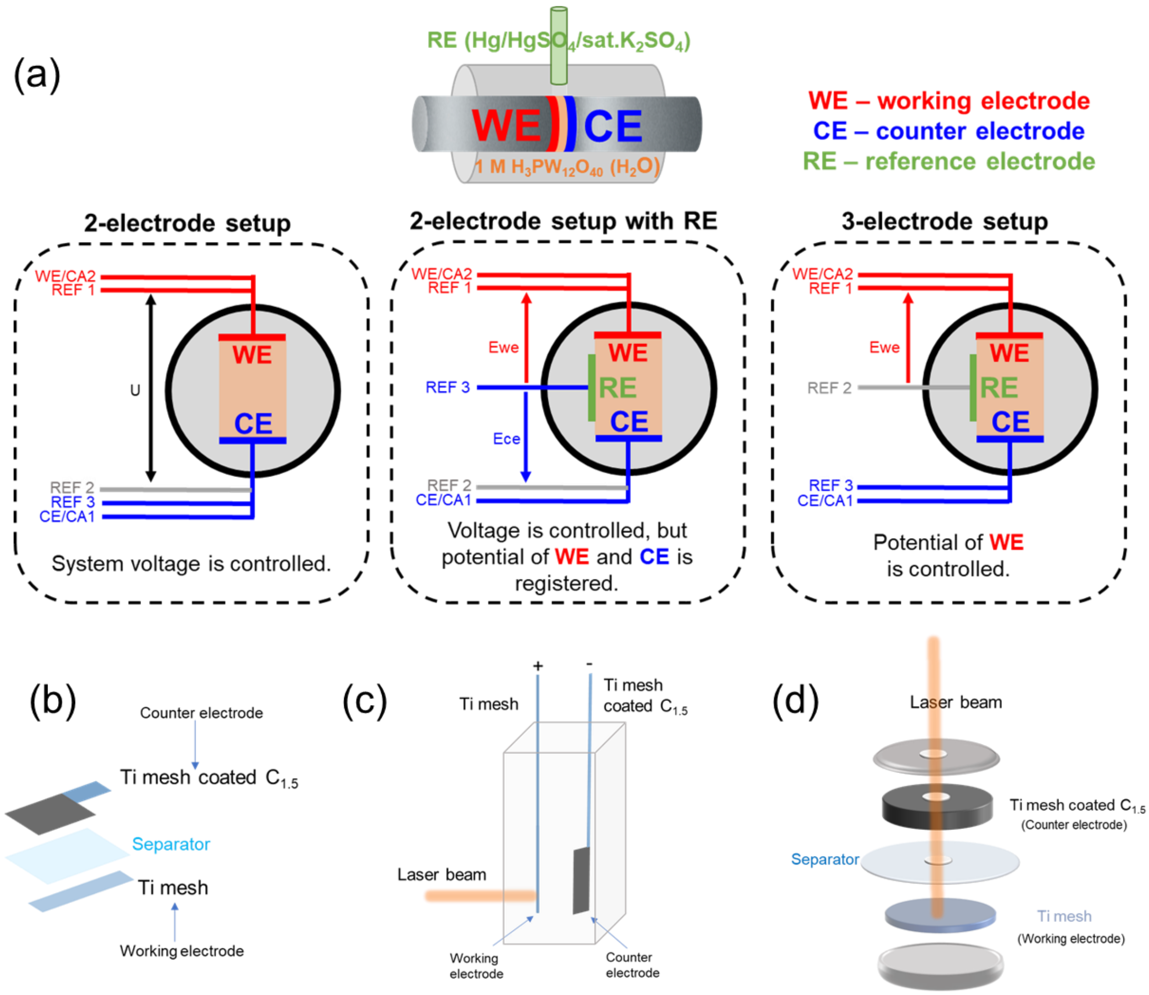

Fig. S3. (a) Schematic representation of electrochemical setups used in conducted experiments based on the homemade cell. (b) Parafilm fixed set-up (active area: WE (0.2 cm*2 cm) and CE (1 cm*2 cm)) for long cycling measurement. (c) UV tube set-up for in-situ Raman and UV measurement (active area: WE (0.1 cm*2 cm) and CE (0.5 cm*2 cm)). (c) For Operando X-ray synchrotron diffraction measurements, dedicated in situ CR2025 coin cells with Kapton windows were used.

7. Density functional theory (DFT) calculations

All first-principles calculations were performed using the Dmol3 module in Materials Studio, with GGA/PBE functional, TS dispersion corrections, and DNP+ basis set.[4,5] The *k*-points of all the molecules were set as Gamma (1 × 1 × 1) in optimization while use 2 × 2 × 1 in single-point energy calculation, and the convergence tolerance was set as 1.0×10^{-5} Ha, 2.0×10^{-3} Ha/Å $^{-1}$, and 5.0×10^{-3} Å for energy, maximum force, and maximum displacement, respectively. The solvent energy was calculated by the **Eq. S1**.

$$E_{ads} = E_{total} - E_{unit} - E_{slab} \tag{S1}$$

Where E_{total}, E_{unit}, E_{slab}, and E_{ads} were the energy of total structure, PWA, carbon (graphite, 001) or Ti (101) substructure, and adsorption.[4,5]

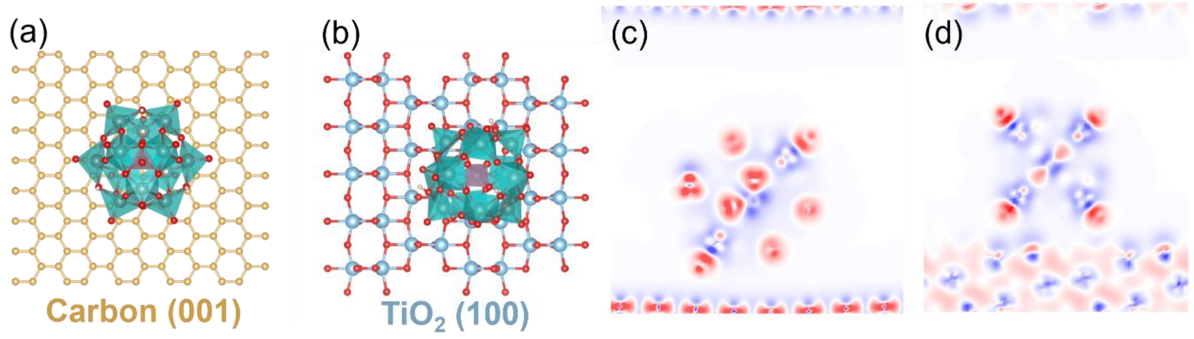

Fig. S4. Top view of Keggin molecule on (**a**) graphite and (**b**) Ti surface. The optimized charge-density-difference patterns of (**c**) Ti (101) and (**d**) graphite (001).

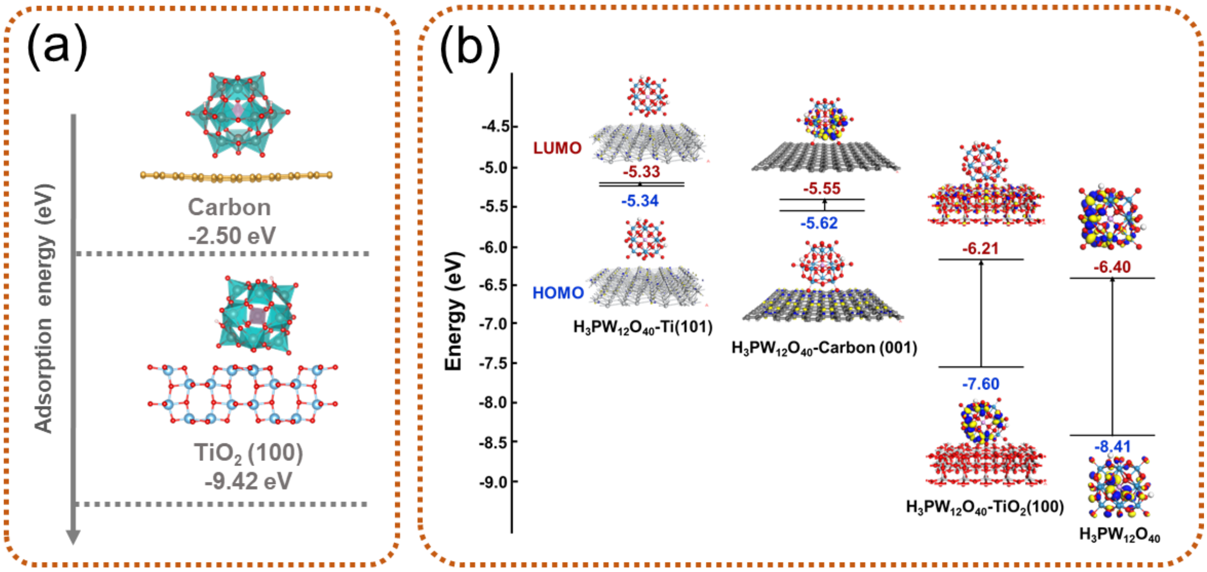

Fig. S5. (**a**) Adsorption energy differences for the interface of graphite and Ti (with home view). (**b**) The HOMO and LUMO of PWA–Ti (101), PWA–Carbon (001), PWA–TiO_2 (100), and PWA systems.

Additionally, DFT calculations (experimental details can be found in section 7 in SI) show that Keggin molecules are more willing to be adsorbed on the TiO_2 surface than on carbon material (graphite). The adsorption energies of Keggin molecules on carbon and TiO_2 were calculated (**Eq. S1**) to be -2.5 and -9.42 eV, respectively (**Fig. S4a, b**). This surface effect of the TiO_2 electrode, next to the achieved redox potential (-0.42 V *vs.* RE) plays a role for the unidirectional charge storage function in the proposed redox CAPode. Furthermore, the optimized charge-density-difference patterns for the adsorption of Keggin molecules on TiO_2 and carbon (graphite) confirm charge transfer between Keggin and Ti atoms, with electron depletion (blue region in **Fig. S4c, d**) and electron accumulation (red region). On the contrary, carbon is minimally affected because of weak adsorption of Keggin on its surface. This Keggin adsorption on the TiO_2 electrode may contribute to the CAPode with high rectification ration. In addition, the LUMO and HOMO of PWA–Ti (101), PWA–Carbon (001), PWA–TiO_2 (100) and PWA systems are calculated based on molecular orbital theory. The equivalent surfaces of HOMO and LUMO and related energies are shown in **Fig. S5**. The lower LUMO energy level means the strong reduction driving force, resulting in highly efficient electron transfer of molecular. The relatively low LUMO of PWA (-6.40 eV) shows that the molecule (or anion) is easily reduced. However, the system of PWA–TiO_2 (100) has the lowest energy value of LUMO (-6.21 eV) when compared to the

other two systems, indicating that the TiO_2 possesses stronger reduction capacity than Ti and carbon for W^{6+} into lower valences.

8. Concentration impact

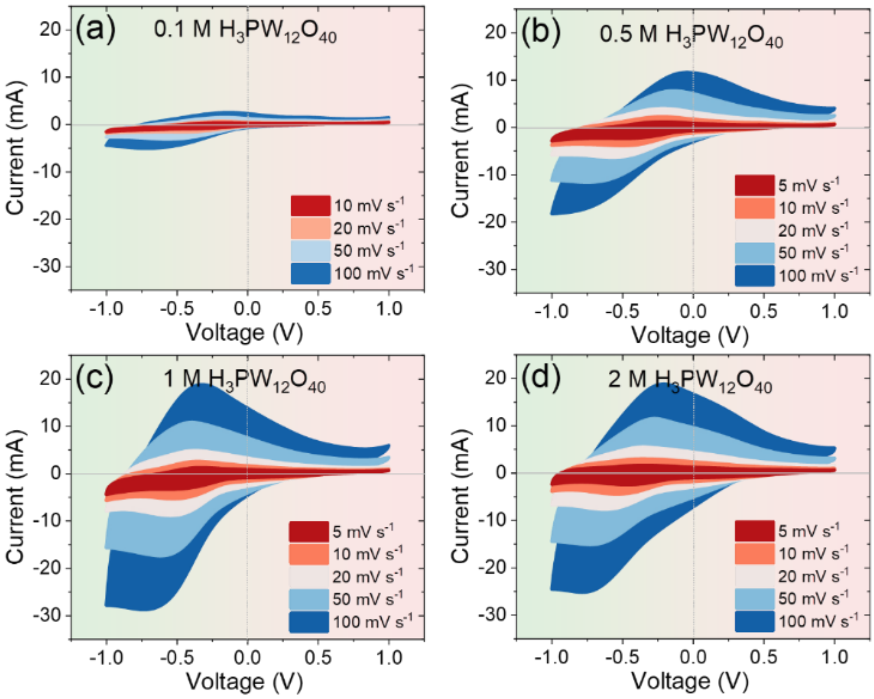

Fig. S6. CV curves for (Ti I PWA I $C_{1.5}$) CAPodes with (**a**) 0.1, (**b**) 0.5, (**c**) 1, and (**d**) 2 M (in H_2O) concentrated electrolyte.

With an increasing concentration of the electrolyte, the current response rises under “blocked” polarization, which results from the presence of more species that can participate in redox reaction and EDL formation. Systems with 1 and 2 M electrolytes show comparable results, however the 1 M-based system has lower current under “blocked” polarization, which is desirable for the CAPode characteristic. This small difference probably comes from the changes in solvation degree at lower pH value, which affects the redox reactions.

9. Details of charge storage mechanism

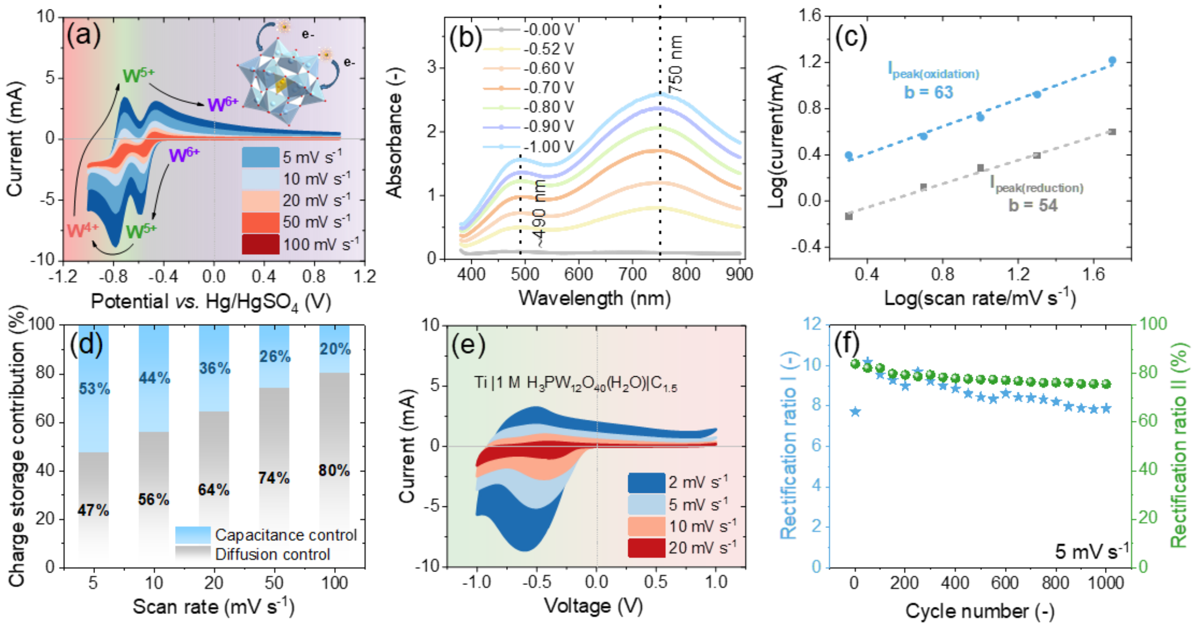

Fig. S7. (**a**) The CV curves for Ti I 1 M PWA, Hg/Hg_2SO_4 reference I $C_{1.5}$. (**b**) UV-spectra registered after different time of cycling applied by CV measurement (5 mV s^{-1}). (**c**) The variation of the *b* value as a function of potential *vs.* RE. (**d**) The diffusion-controlled and surface capacitive contributions to the total stored charge. (**e**) CV curves for (Ti I 1 M PWA I $C_{1.5}$) CAPode. (**f**) RR_I and RR_{II} changes with cycle number applied by CV measurement.

The two reduction peaks at -0.55 and -0.75 V *vs.* RE and two oxidation peaks at -0.49 and -0.72 V *vs.* RE correspond to the surface redox $W^{6+} \leftrightarrow W^{5+} \leftrightarrow W^{4+}$ reaction (**Fig. S7a**). As it was already mentioned, **Eq. 1** presents the first stage of reduction/oxidation ($W^{6+} \leftrightarrow W^{5+}$) of the Keggin-{PW12} POM. The second stage of reduction/oxidation ($W^{5+} \leftrightarrow W^{4+}$; **Eq. S2**) is not achieved in the CAPode when 1 V is applied.

$$PW_{12}^{6+}O_{40}{}^{3-} + 2e^{-} \leftrightharpoons PW_{11}^{6+}W^{4+}O_{40}{}^{5-} \quad \text{(S2)}$$

As the scan rate increases, the current response under “open” polarization gradually increases, while there is almost no current response under “blocked” polarization (the inevitable presence of a small current response is caused by the EDL formation of the $PW_{12}O_{40}{}^{3-}$ anions on the Ti electrode with small surface area).

Fig. S7b presets UV-spectra registered after different time of cycling applied by CV measurement (at 5 mV s^{-1}). Scans taken for the redox reductions of 10 mM PWA in H_2O as the electrolyte solution. The working electrode was a Ti wire. The counter electrode was loaded with $C_{1.5}$ in titanium mesh and the reference mercury, containing a saturated aqueous solution of mercury chloride. Reference and counter electrodes were separated in the reaction mixture by a porous glass frit. Applying a potential of -0.52 and -1 V with the set-up shown and discussed in **Fig. S7b**. Scans were taken every 10 min until the convergence point was reached; once achieved, the potential was set at 0 V.

From the UV/Vis-spectra in **Fig. S7b**, it can be clearly seen that the W^{5+} corresponds to the band registered at 750 nm and at ~500 nm, when potential applied from -0.52 to -1.0 V, whereas there is no such absorption characteristics without applied potential. A general approach for analyzing the electrochemical kinetic processes, based on the scan rate test data, is described by **Eq. S3**.

$$\mathrm{i} = \mathrm{av}^{\mathrm{b}} \tag{S3}$$

where *a* and *b* are adjustable parameters, *I* is the current (A), and *v* is the scan rate (V s^{-1}).

Fig. S7c shows the variation of the *b* value as a function of potential *vs.* RE (**Eq. S3**). Generally, the coefficient *b* varies in the range 0.5 – 1 and the *b*-value of 0.5 represents a diffusion-limited process, while 1 indicates a capacitive process. The *b*-value determined by the slopes of the four redox peaks are 0.89, 0.75, 0.92, and 0.78, which implies that the capacity of VS_2 is little influenced by the diffusion process. Furthermore, the relationship $I = av^b$ can be divided into two parts including capacitive (k_1v) and diffusion-limited effects ($k_2v^{1/2}$), as described by **Eq. S4**.

$$\mathrm{i} = \mathrm{k_1 v} + \mathrm{k_2 v^{0.5}} \tag{S4}$$

or

$$\frac{\mathrm{i}}{\mathrm{v^{0.5}}} = \mathrm{k_1 v^{0.5}} + \mathrm{k_2} \tag{S5}$$

At a particular voltage, the k_1-value characterizes the capacitive and k_2-value corresponds to diffusion limited.[6,7]

It can be seen that b values are in the range of $0.5 < b < 1.0$, indicating a capacitive process mainly due to surface redox reactions and EDL. To study the kinetics behavior, diffusion-controlled and surface capacitive contributions to the total stored charge at the Ti electrode at different scan rates were analyzed according to Dunn's method.[6] As shown in **Fig. S7d**, 47% of the total capacity is contributed by the diffusion processes at 5 mV s^{-1}, and this gradually increases to 80% at 100 mV s^{-1}, verifying the fast reaction kinetics on the Ti electrode (**Eq. S4** and **S5**).

Fig. S7e shows CV curves of the CAPode at various scan rates within a suitable operating voltage window of +/-1.0 V. The CAPode exhibits an obvious asymmetric CV curve shape with an almost coincident "blocking" voltage point (near 0 V) at varying scan rates (2 – 100 mV s^{-1}).

As shown in **Fig. S7f**, the RR_I (explained in next section) stabilizes at about 8 after 1000 cycles and the RR_{II} (explained in next section) keeps constant around 80%. Notably, a redox peak appears under "blocked" polarization of the CV curve after the cycling test (**Fig. S8**, which may be attributed to the change of overpotential caused by the change in pH.

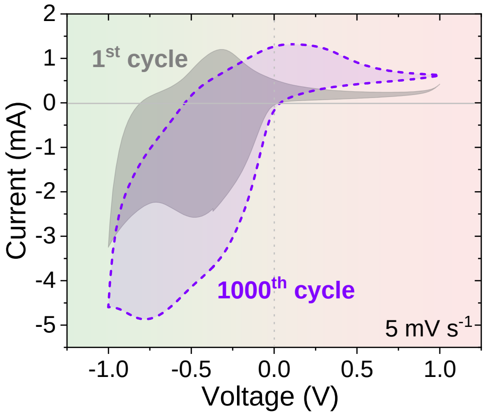

Fig. S8. CV curves (5 mV s^{-1}) registered for (Ti I 1 M PWA I $C_{1.5}$) CAPode system for 1st cycle and 1000 th cycle.

The individual scan rates applied in the CV technique for the Ti(-) and C1.5(+) electrodes (measured in a 3-electrode setup; **Fig. 2b**) were calculated based on the discharge GCD profiles shown in **Fig. 2a** (measured in a 2-electrode setup with a RE). By dividing the working potential range of each electrode by its discharge time, the scan rate corresponding to the application of the current density in the GCD technique can be determined. The calculated scan rates for Ti(-) and $C_{1.5}$(+) are 2.6 and 4.8 mV s^{-1}, respectively, corresponding to the discharge of the system at a current density of 0.5 A g^{-1} within the "open" polarization range from OCV (-0.16 V).

10. Rectification ratios

Rectification ratio I (RR_I):

$$RR_I = \frac{I_1}{I_2} \qquad \text{(S6)}$$

Where: I_1 and I_2 are the currents (A) registered at specified voltage (most often at the highest applied; here ±1.5 V) under “open” and “blocked” polarization, respectively.

Rectification ratio II (RR_{II}):

$$RR_{II} = \frac{C_{spec,O}}{C_{spec,B}} \qquad \text{(S7)}$$

Where: $C_{spec,O}$ is the gravimetric capacitance (F g^{-1}) under “open” conditions, $C_{spec,\,B}$ is the cumulative gravimetric capacitance (F g^{-1}) under “open” and “blocked” conditions.

The gravimetric capacitance of the system is determined via **Eq. S8**.

$$C_{spec} = \frac{1}{v\,\backslash(V_2 - V_1\backslash)m} \int_{V_1}^{V_2} I\,dV \qquad \text{(S8)}$$

Where: C_{spec} is the integral gravimetric capacitance (F g^{-1}) for a single electrode, v is the potential scan rate (mV s^{-1}), m is the mass of electroactive materials of a single electrode and I is the current response during the cycling (for the case of CAPode; the mass of electroactive materials of the working electrode is used).[5,6] For the sake of capacitance calculations, the voltage range $V_2 - V_1$ is used. Four cycles of the electrode and four sets of measurements were carried out before any analysis was performed. Prior to the data analysis, the electrode was cycled 4 times and each measurement was repeated 4 times.[8, 9]

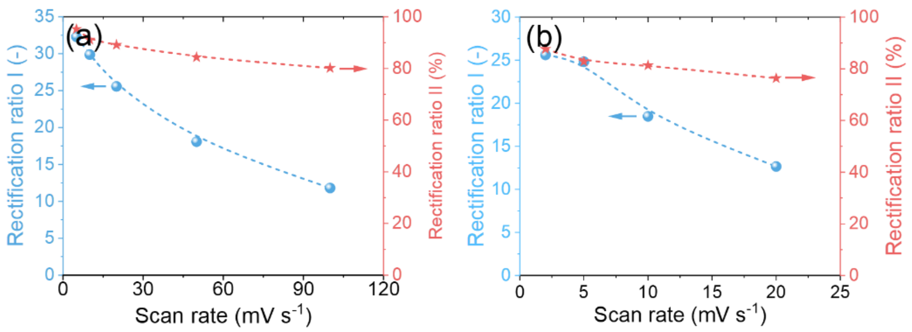

Fig. S9. RR_I and RR_{II} changes for (**a**) 2- and (**b**) 3-electrode setup (with 1 M $H_3PW_{12}O_4$ in H_2O) depending on the applied scan rate.

Interestingly, RR_I and RR_{II} (at ±1.0 V) reach values up to 32 and 95% for 2-electrode setup (**Fig. S9a**) and 26 and 85% for 3-electrode setup (**Fig. S9b**), and they decrease slightly with increasing scan rates. This may result from the sluggish kinetics of redox reactions for the electrolyte.

11. Symmetric systems

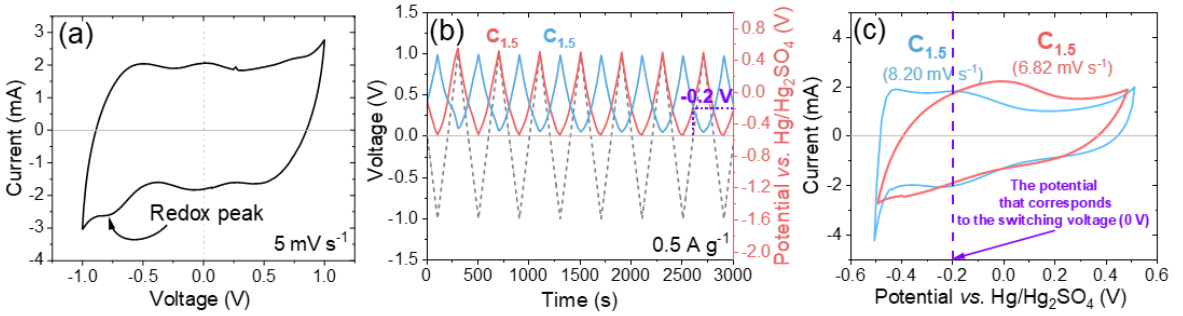

Fig. S10. (**a**) The GCD profiles registered in 2-electrode setup with RE, (**b**) CV curves registered in 3-electrode setup, and (**c**) CV curve registered in 2-electrode setup for ($C_{1.5}$ I 1 M $H_3PW_{12}O_4$ I $C_{1.5}$) symmetric system.

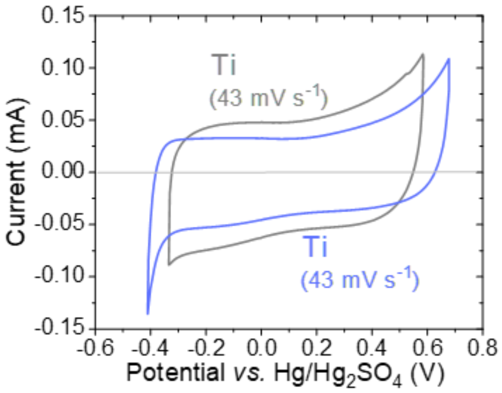

Fig. S11. The CV curves registered in 3-electrode setup for (Ti I 1 M PWA I Ti) CAPode.

The minor difference on the potential range of two electrodes may result from the slight changes on the surface of TiO_2.

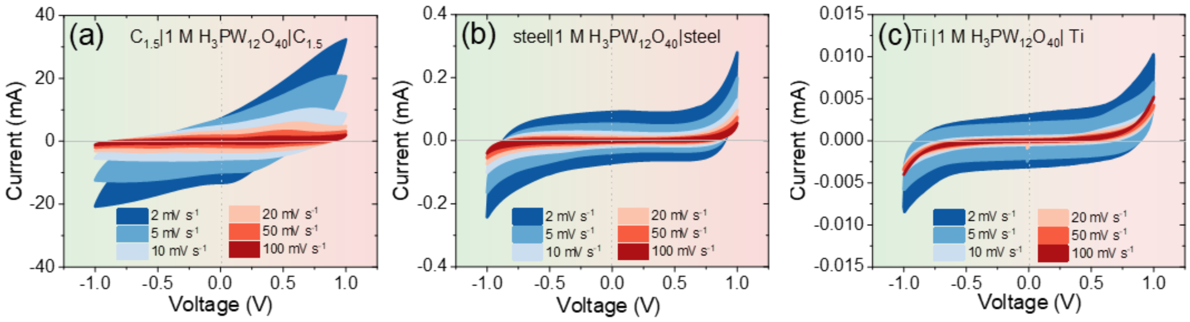

Fig. S12. CV curves registered for symmetric cells: (**a**) ($C_{1.5}$ I 1 M PWA I $C_{1.5}$), (**b**) (steel I 1 M PWA I steel), and (**c**) (Ti I 1 M PWA I Ti).

12. MAS NMR spectroscopy

^{31}P magic angle spinning nuclear magnetic resonance (MAS NMR) spectra were obtained using a commercially available solid-state 2.5 mm double resonance (^{1}H, X) MAS NMR probe in a Bruker Avance Neo spectrometer (Bruker Biospin; Karlsruhe; Germany) tuned to an operating frequency of 121.49 MHz. The sample rotation was set to 15 kHz and chemical shifts were referenced to TMS using phosphorylated serine as a secondary reference (0.3 ppm relative to TMS). Sample loading was performed by the incipient wetness method in an Ar-filled environment ($x < 0.1$ ppm H_2O and O_2). Carbon was loaded with a solution of 1 M PWA in deuterated water (d-H_2O) corresponding to two times the pore volume, as determined from the N_2-adsorption isotherms. The resulting mixture was mortared for 120 s.

For the carbon material, three signals occur which are assigned to ions in the free bulk (1), in contact with the outer particle service (2) and adsorbed inside the pores (3), see also the inserted signal decomposition and the sketch (the inset plot in **Fig.2e**).

In addition to the main signals of the large keggin ions discussed in the manuscript, there is a minor signal in pure solution at 0.3 ppm (**Fig. S14**). This chemical shift is characteristic for phosphoric acid/phosphate, which means that the solution obviously contains a minor amount of phosphoric acid/ phosphate. Note that this minor signal represents less than 5% of the total ^{31}P signal intensity. Our experimental conditions, particularly the very acidic environment (pH < 1), are highly favorable for maintaining the stability of PWA. Literature strongly supports the fact that phosphotungstic acid is stable under such acidic conditions and does not readily decompose into H_3PO_4 or other phosphate species unless subjected to extreme reductive potentials over an extended period, which were not encountered in our experimental setup.[10] However, the synthesis of phosphotungstic acid typically involves the reaction of sodium tungstate and phosphoric acid, and residual unreacted phosphoric acid or phosphate ions (PO_4^{3-}) are common impurities, even in high-purity preparations of PWA. Therefore, the presence of phosphoric acid/ phosphate in PWA may come from incomplete formation or minor impurities during the synthesis process. This signal at 0.3 ppm in the bulk solution is shifted by -6.2 ppm for the electrolyte-loaded carbon-material, i.e., this minority species is also taken up by the pore system.

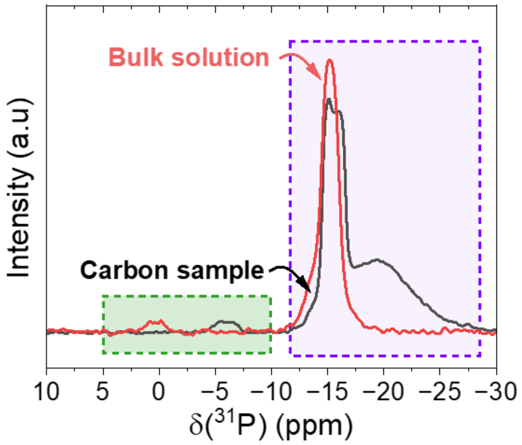

Fig. S13. ^{31}P MAS NMR spectra of pure bulk 1 M PWA in d-H_2O electrolyte solution (red) and the $C_{1.5}$ electrode material (black) loaded with electrolyte (loaded electrolyte volume = two times the absolute pore volume).

13. The role of electrolyte

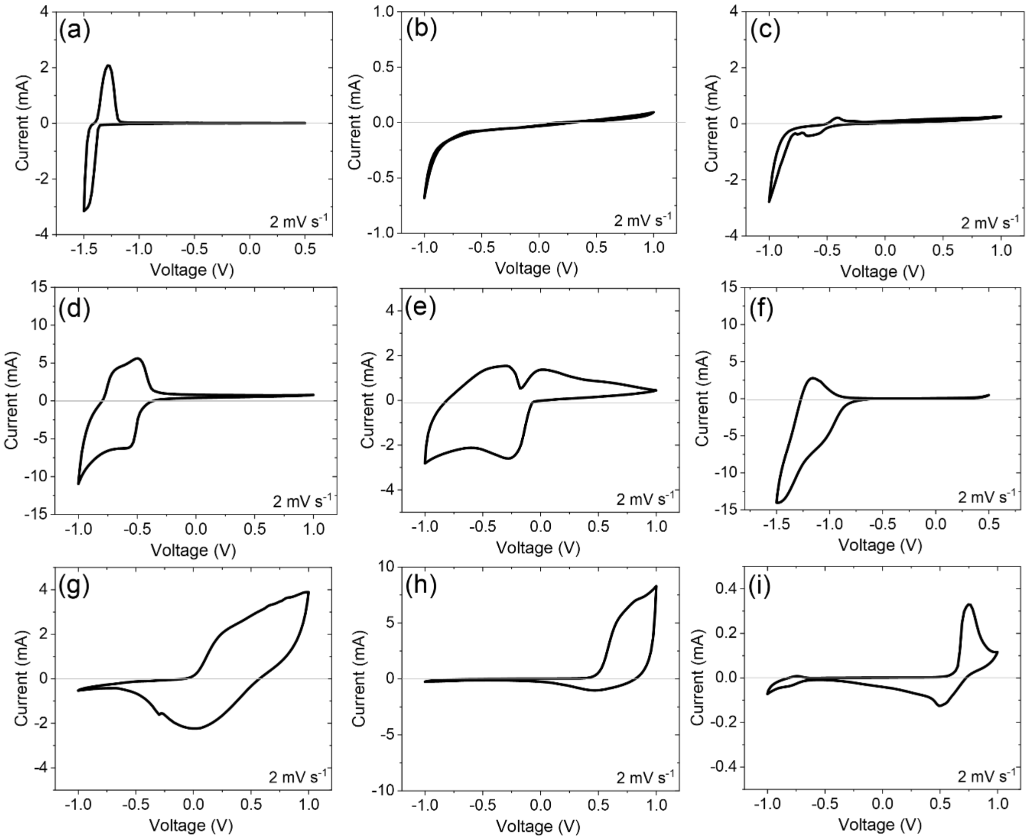

Fig. S14. CV curves for asymmetric cell of Ti I electrolyte I $C_{1.5}$ based on different redox couples in 1 M various electrolytes in 1 M H_2SO_4 (**a**) $Zn(TFSI)_2$, (**b**) $Cr(NO_3)_3$, (**c**) $Co(NO_3)_2$, (**d**) $CuSO_4$, (**e**) Na_2MoO_4, (**f**) VCl_3, (**g**) $FeCl_2$, (**h**) LiBr, and (**i**) $MnSO_4$.

The thermodynamic dissociation constants and Hammett acidity functions indicate that PWA maintains its strong acidic nature (dissociating in the H_2O as a solvent), which is essential for its performance in electrochemical processes. PWA exhibits high ionic conductivity, which allows for efficient charge transfer and improved CAPode performance, comparable to conventional H_2SO_4-based systems.[11] Moreover, Keggin structure provides a high number of surface-active sites.[12,13] Furthermore, PWA provides higher corrosion resistance compared to conventional acidic electrolytes like H_2SO_4. This property ensures stable performance and longevity, even with low-cost non-noble current collectors.[11] The reduction of W^{6+} to W^{5+} in PWA is a significant process in electrochemical reactions, often accompanied by a visible color change. This color change can be detected using UV-Vis spectroscopy, making it a useful tool for exploring specific reactions in electrochemical processes. This is due to

intervalence charge transfer between W^{5+} and W^{6+} ions, which can be observed as a shift in the UV-Vis spectrum, which is similar with the reduction of W^{6+} to W^{5+} in tungsten-based materials, such as WO_3.[14,15]

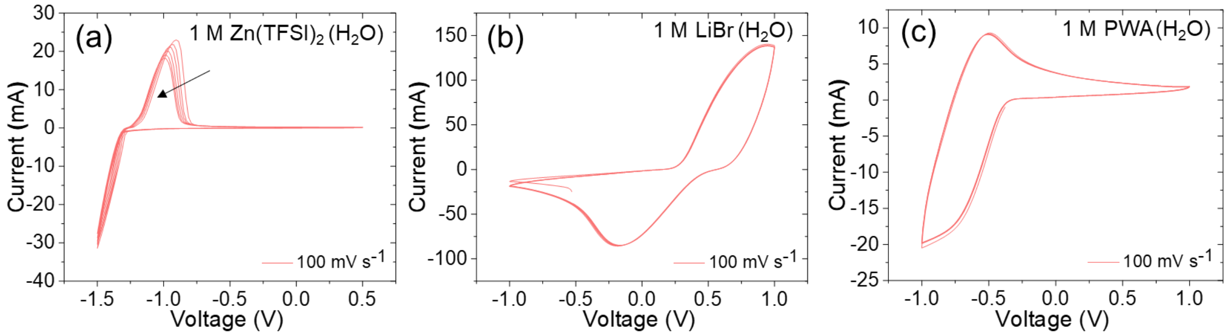

Fig. S15 CV curves registered for 6 cycles at the same scan rate (100 mV s^{-1}) for asymmetric cells (Ti I electrolyte I $C_{1.5}$) based on different 1 M redox couples: (**a**) $Zn(TFSI)_2$ (in H_2O), (**b**) LiBr (in H_2O) (pH adjusted by H_2SO_4), and (**c**) PWA (in H_2O).

To optimize the rectification performance of CAPodes, we dissolved various redox-active salts in 1 M H_2SO_4, except for PWA, which was excluded to maintain pH stability and solubility. The tested salts included $Zn(TFSI)_2$, $Cr(NO_3)_3$, $Co(NO_3)_2$, $CuSO_4$, Na_2MoO_4, VCl_3, $FeCl_2$, LiBr, and $MnSO_4$. Among these, only $Zn(TFSI)_2$, LiBr, and PWA exhibited high rectification ratios (*RR*). In contrast, other CAPode systems demonstrated low *RR* values, likely due to the influence of the electrolyte on electrode polarization, which can induce side reactions such as water decomposition. However, in the $Zn(TFSI)_2$ system, the CAPode mechanism is based on Zn ion plating/stripping (Zn^{2+}/Zn^0) on the surface of the Ti mesh. It is noteworthy that the current response decreases with CV cycles in **Fig. S15a**, which may result from Zn ion plating/stripping (Zn^{2+}/Zn^0) process generating by-products (dendrites) and parasitic reactions that accumulate over time,[16] which negatively impact cycling stability and ultimately damage the rectification performance of the CAPode. Remarkably, the potential of Zn^{2+}/Zn^0 overlap the potential of hydrogen evolution, which also can disrupt the operation of the CAPode. Furthermore, the voltage window in positive and negative range is not symmetrical due to the quite negative potential of Zn ion plating/stripping (Zn^{2+}/Zn^0), which can highly affect the further application of the CAPode in logic gates. Furthermore, in the CAPode system using LiBr as electrolyte, a redox reaction ($2Br^-/Br_2$) occurs. The acidity of the solution plays a crucial role in the stability and

performance of the Br^-/Br_2 redox couple. More acidic solutions broaden the potential range where intermediate bromine species are dominant, which can affect the overall efficiency and stability of the system.[17,18] Also, comparing with the results with a scan rate of 2 mV s^{-1} (**Fig. S14**), it is noteworthy that the difference in the voltage between reduction and oxidation peak is enhanced in CV curves registered at 100 mV s^{-1}, also resulting in a high current response at “negative” voltage, which decreases the rectification performance of this CAPode (**Fig. S15b**). The redox peaks and current response in the negative voltage range remain stable in the CAPode system based on 1 M PMA in H_2O after several cycles (**Fig. S15c**).

14. Rectification mechanism

To understanding of the rectification mechanism of the CAPode with Keggin electrolyte, we investigated its charge storage kinetics using *ex* and *in situ*-measurements with a customized 2-electrode cell (**Fig. S16**).

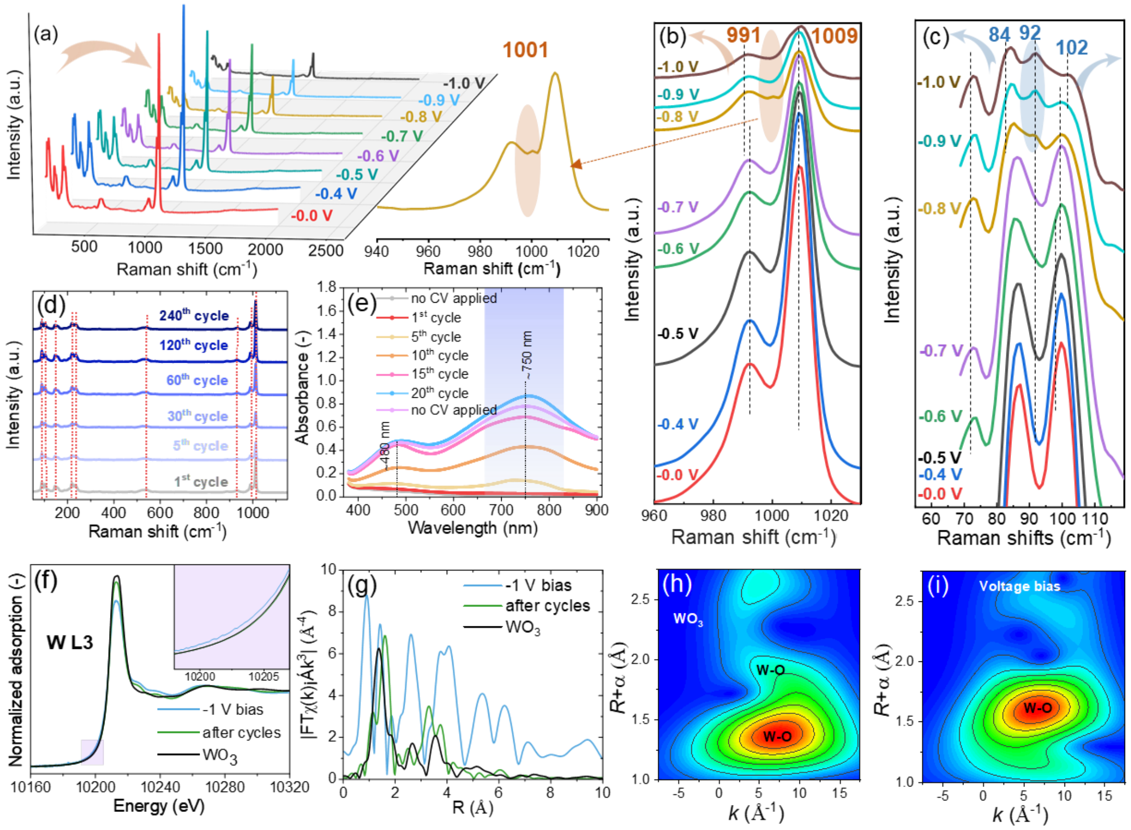

Fig. S16. (**a**) *In situ*-Raman spectra of Ti I 1 M PWA I $C_{1.5}$ under different voltages. (**b, c**) Enlarged *in situ*-Raman spectra from **Fig. S16a** at relevant wavelength ranges. (**d**) *Ex-situ* Raman spectra of Ti I 1 M PWA I $C_{1.5}$ after different CV cycles. (**e**) *In situ*-Vis-spectra of Ti I 10 mM PWA I $C_{1.5}$ by applying a CV (5 mV s^{-1}). (**f**) W L3-edge XANES experimental spectra of WO_3, the PWA without voltage and with voltage bias. (**g**) FT-EXAFS fitting curves at R space of the Sn K-edge of WO_3, the PWA with -1 V bias and after cycles. (**h, i**) WT-EXAFS signals the k^2-weighted EXAFS spectrum of WO_3 and the PWA with voltage bias, respectively.

In situ-Raman measurements were used to monitor the structural changes of $H_3PW_{12}O_{40}$ caused by the redox reaction. **Fig. S16a** displays the characteristic peaks of the Keggin skeleton. The weaker Raman bands at 890 and 530 cm^{-1} are characteristic of the asymmetric stretching vibration of bridging W−O_b−W (O_b denotes

a corner-sharing bridging oxygen atom) and the symmetric stretching of bridging W−O_c−W (O_c denotes an edge-sharing bridging oxygen atom), respectively.[19] The enlarged Raman spectra in **Fig. S16b** show strong W-O symmetric and asymmetric stretching modes at symmetric (v_s) stretching modes of W–O_d ((W–O_d)v_s, 1009 cm^{-1}) and anti-symmetric (v_{as}) ((W–O_d)v_{as}, 992 cm^{-1}), respectively.[20] The asymmetric stretch is a consequence of the presence of vibrationally coupled adjacent W-O_d bonds in the Keggin structure. However, a shift of (W–O_d) v_{as} appears at 991 cm^{-1} accompanied by a new small band at 1001 cm^{-1} (-0.8 V). The Raman band at 80~100 cm^{-1} represents the bending mode of the bridging W−O_c−W bonds of the intact Keggin structure (**Fig. S16c**).[21] It is noteworthy that a new bulky band appears at 92 cm^{-1} from the -0.8 V applied voltage accompanied by two bands showing relatively large shift to 84 and 102 cm^{-1}, which may result from a greater (less regular) distortion of the octahedral WO_6 units due to the electron transfer in redox processes.[21,22] The changes of subordinate peaks for these bridging bands may result from the electron oxidation and reduction process on the W atom.[22] The significant decrease in Raman peak intensity at -1 V can be primarily due to changes in the electronic interactions and structural transformations of the chemisorbed molecules on the electrode surface.[23] **Fig. S16d** shows the Raman spectrum of the electrolyte $H_3PW_{12}O_{40}$ (lower spectrum). After 48h, there are no changes for the Keggin skeleton characteristic peaks ascribed to the symmetric-asymmetric stretching modes of (W–O_d)v_s (1011 cm^{-1}), (W–O_d)v_{as} (994 cm^{-1}), P–O_a (906 cm^{-1}), W-O_b-W (522 cm^{-1}), and W-O_c-W (237 cm^{-1}) bonds (**Fig. 1b**). These confirm the results of stability performance after 1000 cycles (**Fig. S8**).[21] **Fig. S16e** shows absorption patterns for the valence of W^{5+} (750 and ~500 nm).[24] The cell with {PW12} solution had a potential of -0.52 V *vs.* RE applied, which was chosen from CV scans as a point exceeding the 1st electron reduction potential (**Eq. 1**), but there is no characteristic absorption in the spectrum of the pristine {PW12} solution. Especially, bands forming at 750 and ~500 nm (corresponding to 1st reduction) become weaker when the CV measurement stops. Close monitoring of these processes allowed for the identification of the absorption profiles and the shift in peaks between the redox processes, making recognition of the reduction process by the UV-vis band possible.

The chemical state and coordination environment surrounding the W atoms in the Keggin structure were analyzed by *ex* and *in situ*-XAS (experimental details can be found in section 14 in SI). The spectra of W *L3*-edge X-ray absorption near-edge

structure (XANES) disclosed that the spectrum for PWA under voltage bias (10199.97 eV) was lower than that for WO_3 (10201.30 eV) (**Fig. S16f** and **S17**), implying that the oxidation states of W species were ≤ W^{6+}. Also, the spectrum of PWA without voltage bias is situated near WO_3 spectrum, indicating the oxidation states equal to W^{6+} (**Fig. S18**). To determine the exact valence state of W in PWA with voltage bias, the data of all samples by XANES were fitted using the linear combination fitting (LCF) method (**Tab. S2**).[25] From the comparison of R-edge (**Fig. S16g**) and the fitting results (**Fig. S19**; **Tab. S2**), it can be seen that the samples mainly contain W-O coordination, and the number of coordination is close to 6.[26,27]

Detailed local coordination structure of the W sites in PWA with voltage bias was obtained by Fourier-transformed (FT) k^2-weighted extended XAFS (FT-EXAFS) analyses. As displayed in the FT-EXAFS spectra of the W *K*-edge, the PWA with voltage bias sample displayed one sharp peak belonging to the scattering path of W−O coordination. Additionally, in comparison with WO_3, there mainly exists a W-O coordination mode, the specific fitting data are shown in **Fig. S20** and **Tab. S2**, and the data are well-fitted from the *k*-space.[28] Different colors represent the height of the peaks, which can not only distinguish the distance of the coordinating atoms (i.e., bond length), but also the type of the coordinating atoms (qualitatively, the higher the atomic number, the more rightward the position of the peaks) (**Fig. S16, i**). Wavelet data combined with the information of the *R*-edge fitting clearly distinguish the sample coordination, and from the small-wavelet analysis. It can be seen that the samples mainly contain W-O coordination, compared to the WO_3 standard samples, which mainly contain a lower proportion of a W-O coordination mode.[29]

Synchrotron X-ray diffraction and adsorption spectroscopy measurements: Operando X-ray synchrotron diffraction measurements were performed at beamline BL04 at the ALBA cells synchrotron (Spain), while *ex situ*-X-ray absorption spectroscopic studies (XAS) were performed at the P64 beamline at PETRA III, German Electron Synchrotron (DESY; Hamburg). For measurements, dedicated in situ CR2025 coin cells with Kapton windows were used. The working electrode was composed of 70 wt% active materials, 15 wt% Super C65 and 15 wt% polytetrafluorethylene (PTFE; Sigma-Aldrich®) pressed directly on a copper foil (Ø = 12 mm) with a loading range of around 3 – 8 mg cm^{-2}.

Data reduction, data analysis, and EXAFS fitting were performed and analyzed with the Athena and Artemis programs of the Demeter data analysis packages that utilize the FEFF6 program to fit EXAFS data.[30, 31] The energy calibration of the sample was conducted through standard and W foil, which as a reference was simultaneously measured. A linear function was subtracted from the pre-edge region, then the edge jump was normalized using Athena software. The $\chi(k)$ data were isolated by subtracting a smooth third-order polynomial approximating the absorption background of an isolated atom. The k3-weighted χ(k) data were Fourier transformed after applying a Hanning window function (Δk = 1). For EXAFS modeling, the global amplitude EXAFS (*CN*, *R*, *σ*2, and *ΔE*0) was obtained by nonlinear fitting, with least-squares refinement, of the EXAFS equation to the Fourier-transformed data in *R*-space, using Artemis software, and the EXAFS of the WO_3 are fitted and the obtained amplitude reduction factor S02 value (0.852) was set in the EXAFS analysis to determine coordination numbers (CNs) in the sample.

For the Wavelet Transform analysis, the $\chi(k)$ exported from Athena was imported into the Hama Fortran code. Parameters were listed as follows: R = 1 – 4 Å, k = 0 – 12 Å^{-1}; k weight = 3; and Morlet function with κ = 15, σ = 1 was used as the mother wavelet to provide the overall distribution.

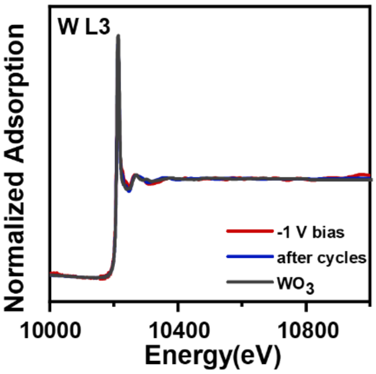

Fig. S17. W L3-edge experimental spectra of WO_3, the PWA without voltage bias and with voltage bias.

Expanding the range would offer finer detail, which is presented in **Fig. S19**. Our primary focus was on the near-edge region (**Fig. 3f**) to capture the oxidation states effectively, and this range was selected based on initial assessments of signal relevance for the redox processes we are studying.

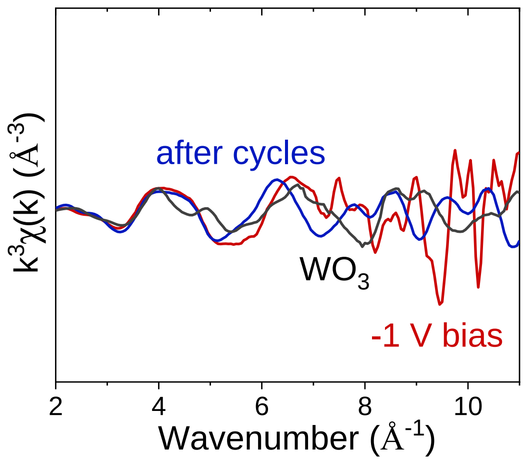

Fig. S18. W K-edge EXAFS fitting curve of WO_3 and PWA before and after cycling (CA; -1 V).

Tab. S2. EXAFS fitting parameters at the W L3–edge for various samples.

Sample	Shell	*can*	R(Å)^b	σ^2(Å^2)^c	ΔE_0(eV)^d	*R* factor
WO_3	W-O	4*	1.78 ± 0.01	0.0069	1.5	0.0165
	W-O	2*	2.12 ± 0.01	0.0061	7.3	
**W sample**	**W-O**	**6.3±0.8**	**2.16 ± 0.01**	**0.0040**	**-11.2**	**0.0195**

^{a}*CN* – coordination number; ^{b}*R* – distance between absorber and backscatter atoms; $^c\sigma^2$ – Debye-Waller factor to account for both thermal and structural disorders; $^d\Delta E_0$ – inner potential correction; *R* – factor indicates the goodness of the fit. S_0^2 was fixed to 0.852, according to the experimental EXAFS fit of WO_3 by fixing CN as the known crystallographic value. A reasonable range of EXAFS fitting parameters: $0.600 < S_0^2 < 1.000$; $CN > 0$; $\sigma^2 > 0$ Å^2; $|\Delta E_0| < 15$ eV; *R* factor < 0.02.

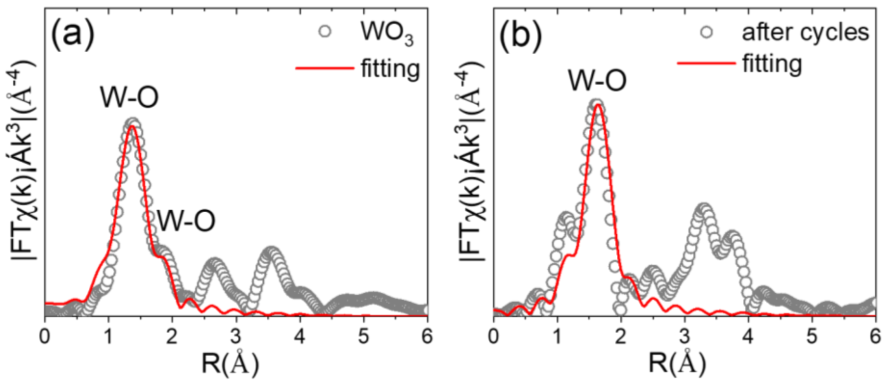

Fig. S19. FT-EXAFS fitting curves at R space of W K-edge of (**a**) WO_3 and (**b**) PWA after cycling.

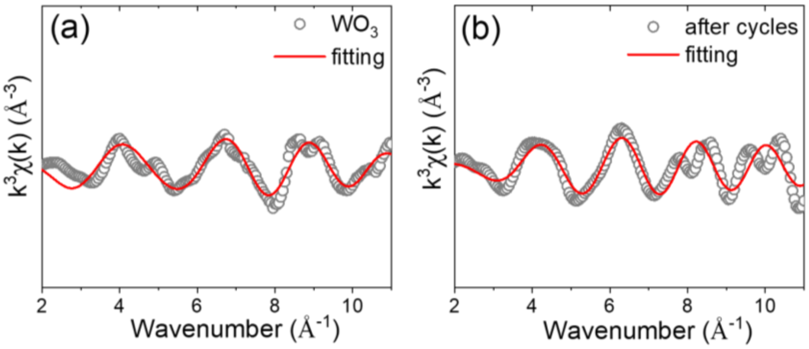

Fig. S20. W K-edge EXAFS fitting curve of (**a**) WO_3 and (**b**) PWA (CA; -1 V) after cycling.

15. Logic gates

Screen printing:

Electrode ink: The electrodes were prepared by mixing 80 wt% $C_{1.5}$ or titanium powder, 10 wt% Super p MA-EN-CO-01 as a conductive agent and 10 wt% PVDF as a binder to yield an ink, using ball milling.

Screen design: Two screens were used, equipped with either a standard PET mesh (150/31; 22.5°) and coated by hand with an all-round photoemulsion (FLX Screen; Siebdruckversand; China). Printed structures are simple lines with line lengths of 12 mm and widths 3.25 mm, and the weight of each electrode is around 3 mg. Furthermore, interdigitated structures with line width and gaps of 100 µm were designed to print micro-ionologic devices. Interdigitated structures with 50 µm gap, which is close to the resolution of the screen-printing method (25 – 30 µm), can be printed but the number of overprint passes can hardly exceed 2 (since by 3 spreading of the ink, fingers will merge). Hence, larger gaps (e.g., 100 – 200 µm) are preferred for overlayer printing. The existence of large particles in the sediment ink is another limiting factor for increasing of the print resolution.

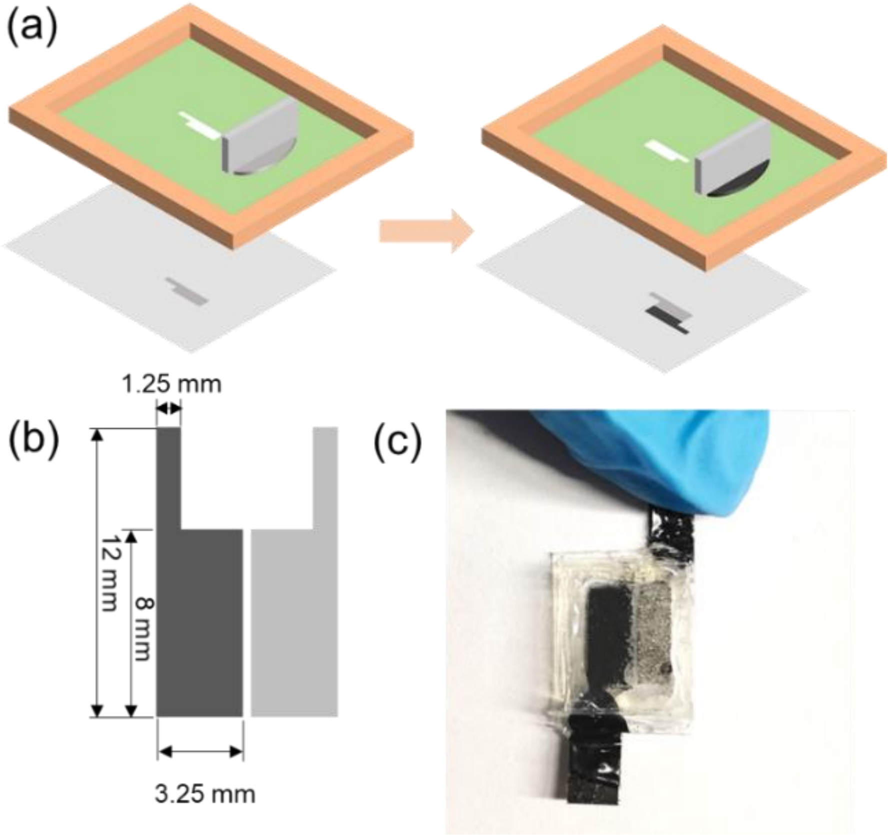

Fig. S21. Monolithic *ip*CAPode architecture: (**a**) scheme of screen-printing method, (**b**) electrodes dimensions, (**c**) photo of the device.

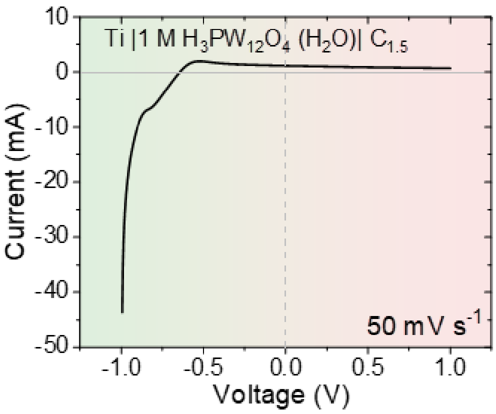

Fig. S22. *I*−*U* curve of the (Ti | 1 M PWA | $C_{1.5}$) CAPode (coin cell).

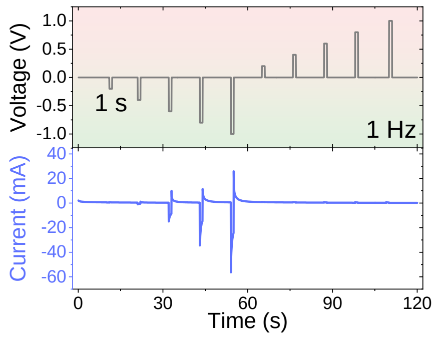

Fig. S23. Current responses of the (Ti | 1 M $H_3PW_{12}O_4$ | $C_{1.5}$) CAPode (coin cell) with gradual (±0.2 V) increase of voltage to ±1 V (1 Hz; controlled by CA).

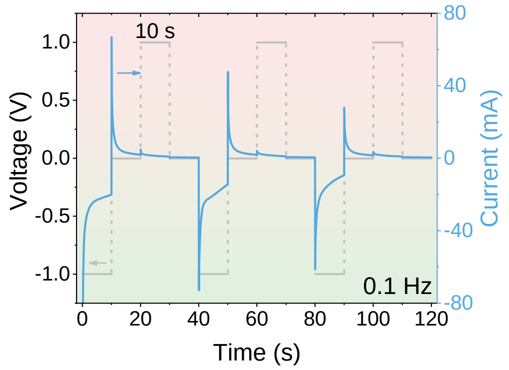

Fig. S24. Current responses of the (Ti | 1 M $H_3PW_{12}O_4$ | $C_{1.5}$) CAPode (coin cell) under a single ±1.0 V pulse (0.1 Hz; controlled by CA).

To evaluate the characteristic response of the (Ti I 1 M PWA I $C_{1.5}$) CAPode, a coin cell was assembled and evaluated for comparing and confirming the same logic responses with a printed cell. In **Fig. S21**, the current of CAPode increases sharply as expected from simple current-voltage (*I-U)* measurements when a "blocked" polarization is applied, whereas then decreases until it saturates. The current on/off response to a single slow voltage pulse (1 Hz; DC bias from ±0.2 to ±1 V) is presented in **Fig. S22**. As the voltage increases, the distinct on/off response is visible, which is even more pronounced when the absolute value of the voltage pulse grows. The current-time (*I*-*t*) curves of the hybrid capacitor diode device at voltages of 1 and -1 V applied alternately (to return their initial states, a 0 V interval was set before each external bias) are depicted in **Fig. S23**.

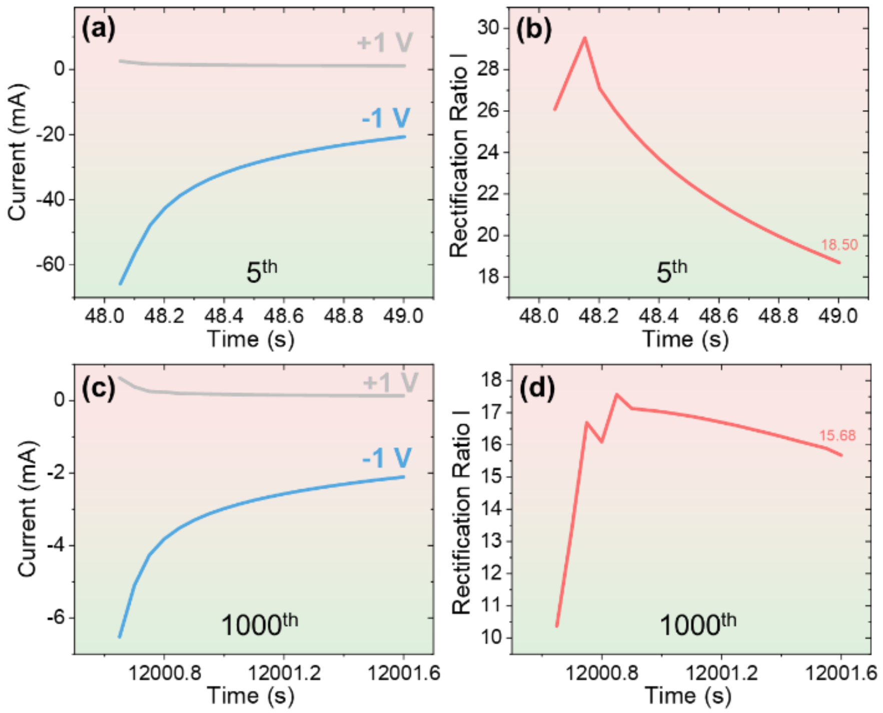

Fig. S25. (**a**) The current responses of the (Ti | 1 M PWA | $C_{1.5}$) CAPode under a single ±1 V pulse (0.1 Hz; controlled by CA) after 5 cycles (for a CAPode, the first several cycles for stabilizing the whole system) and (**b**) the calculated RR_I. (**c**) The current responses of the (Ti | 1 M PWA | $C_{1.5}$) CAPode under a single ±1 V pulse (0.1 Hz; controlled by CA) after 1000 cycles and (**d**) the calculated RR_I.

When different biases ("on" and "off") are applied, the current response at the "off" bias (1 V) drops faster than that at the "on" bias (-1 V). When a longer voltage pulse is applied to the *ip*CAPode (**Fig. S25a**), the behavior is similar, but the RR_I is slightly lower than the initial state and finally stabilizes (**Fig. S25b**). Hence, as the duration of the voltage pulse increases, the rectification ratio changes and finally saturates. We measured key performance metrics such as the on/off current ratio at the voltage of ±1 V (transconductance 1000 cycles) for CAPode (**Fig. S25c and S25d**) in the G-CAPode, which keeps more than 84% of RR_I after 1000 cycles.

The primary factor limiting cycling stability may stem from the cell setup. In this study, we used a parafilm-fixed for long-term cycling, which can lead to solvent evaporation. Since the mechanism described in this work involves the redox-active ions from the electrolyte, solvent retention plays a crucial role in maintaining system stability.

Quantification of Time Constants in CAPode Performance

The performance of CAPode devices is significantly influenced by ion dynamics, which can be quantitatively analyzed through two key parameters: the device time constant (τ_{device}), derived from the Transient Response Method, and the diffusion time constant ($\tau_{\text{diffusion}}$), calculated from ion diffusion coefficients obtained via Electrochemical Impedance Spectroscopy (EIS). These parameters provide insights into the overall charging/discharging behavior and ion transport efficiency within the electrode structure.

(a) Time Constant from the Transient Response Method:

The transient response method measures the CAPode's time-dependent behavior when subjected to a step change in voltage or current. This approach captures the charging and discharging dynamics of the device, with the characteristic τ_{device} extracted by fitting the transient response curve (e.g., current *vs.* time). The response is commonly modeled using an exponential decay function:

$$I\backslash(t\backslash) = I0 \times e^{\frac{-t}{\tau_{device}}} \tag{S9}$$

Where I_0 is the initial current, t is the time, and τ_{device} is the characteristic time constant.[32] **Fig. S25a** illustrates the transient response of the CAPode, from which τ_{device} was determined to be approximately 5.6 s. This value reflects the intrinsic time required for the device to complete its charge-discharge cycle.

(b) Diffusion Time Constant from the Ion Diffusion Coefficient (D):

Ion diffusion plays a critical role in the electrochemical performance of CAPode devices. The $\tau_{\text{diffusion}}$ is calculated as:

$$\tau_{\text{device}} = \frac{L^2}{D} \tag{S10}$$

Where L is the effective diffusion length (the distance over which ions diffuse), and D is the diffusion coefficient of the ions, which can be extracted from the Warburg impedance (σ) obtained from Electrochemical Impedance Spectroscopy (EIS).[33]

We used EIS to derive the Warburg impedance, which is typically represented as:

$$D = \frac{R^2T^2}{2A^2n^4F^4C^2\sigma^2} \quad \text{(S11)}$$

Where R is gas constant (8.314 J mol^{-1} K^{-1}), T is absolute temperature (K), A is electrode area (cm^2), n is number of electrons transferred in the reaction, F is Faraday constant (96485 C mol^{-1}), C is concentration of the diffusing species (mol cm^{-3}), σ is Warburg coefficient, extracted from EIS data based on $Z_{\mathrm{real}} \propto \omega^{-1/2}$.

From the Randles plot of the CAPode (**Fig. S25b)**, the Warburg coefficient σ was determined to be 156.7 Ω s$^{1/2}$. With an electrode area (0.504 cm^2), D is calculated to 5.68 × 10^{-12} cm^2 s^{-1}. L of bulk Ti electrode is an average value (around 10 μm), we calculated the diffusion time constant $\tau_{diffusion}$. The diffusion time constant was calculated to be approximately 176123 s.

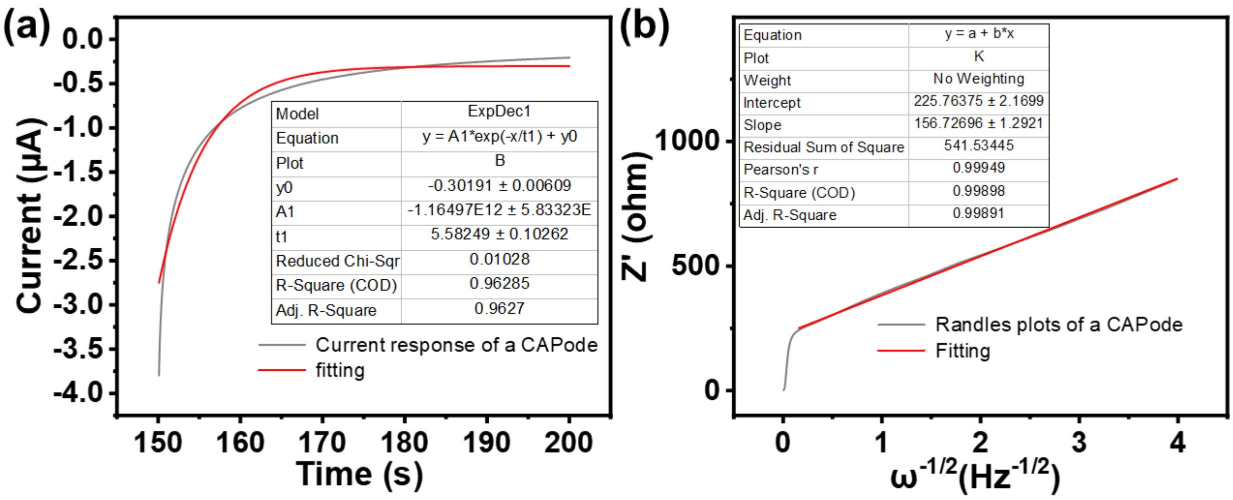

Fig. S26. (**a**) The current response of a bulk CAPode and the related fitted results. (b) The Randles plots of a bulk CAPode and the related fitted results.

In conslusion, due to $\tau_{device} \ll \tau_{diffusion}$, faster processes like electron transfer or interfacial reactions dominate the system's dynamics. Ion diffusion is not a limiting factor in the CAPode's performance.

The input/output signals were measured by using instruments (ROHDE & SCHWARZ RTB2004, KEYSIGHT 33600A, KEYSIGHT B2912A, and KEYSIGHT B2912A) at TU Dresden.

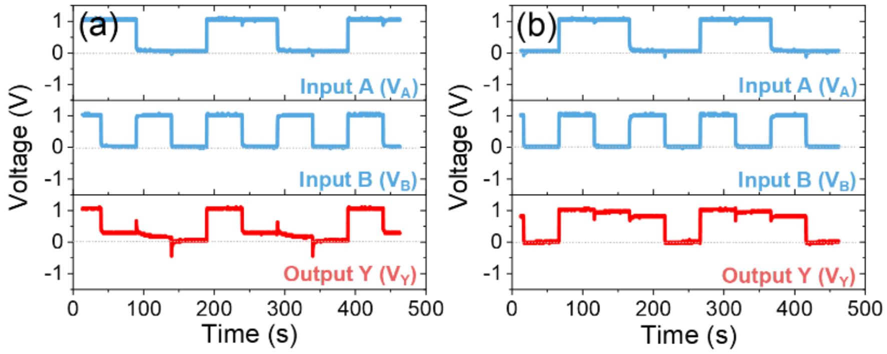

Fig. S27. Input signals of (0,0), (1,0), (0,1), (1,1) and corresponding outputs of coin cells for (**a**) OR and (**b**) AND logic circuits.

The logic function scheme of input signals of (0,0), (1,0), (0,1), and (1,1) of OR and AND based on coin cells is shown in **Fig. S26**, and all the output voltages of "0" and "1" gate are close to the real 0 V and 1 V, respectively. These stunning output signals come from the adjusted resistance of 1 kΩ and current-generated by this circuit here, resulting in a minor IR drop.

Besides the outer resistance and current generated by the circuit, this distortion of "1" also results from the high resistance of the printed CAPodes when it is fully charged and work like a resistor. These output voltage signals also present the characteristics of charging and discharging curves in ECs, as CAPodes devices are based on hybrid ECs.

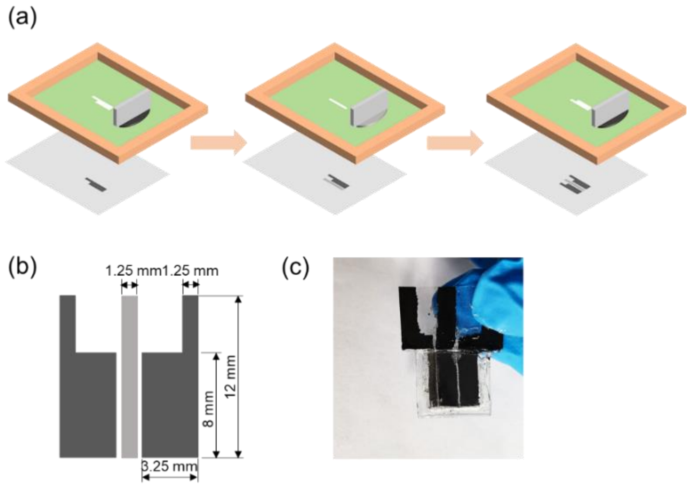

Fig. S28. Monolithic *ip*G-CAPode architecture with GE: (**a**) scheme of screen-printing method. (**b**) electrodes dimensions, (**c**) photo of the device.

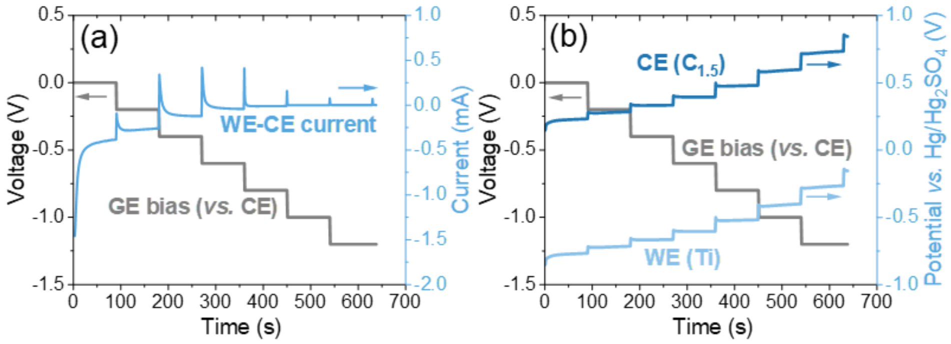

Fig. S29. (**a**) Current response and (**b**) potential changes of CAPode electrodes (WE and CE) with different voltage biases applied to the GE *vs.* CE controlled by CA technique (CAPode: Ti I 1 M PWA I $C_{1.5}$; GE: $C_{1.5}$).

The current change with different voltage biases applied to the gate electrode is presented in **Fig. S29a**. The potential of WE and CE shifts from -0.8 – 0.2 V *vs.* RE to -0.3 – 0.7 V *vs.* RE, respectively when the negative voltage biases from 0 to -1.2 V are applied to GE (**Fig. S29b**), which is caused by the applied voltage on GE driving the working potential shifting WE and CE.

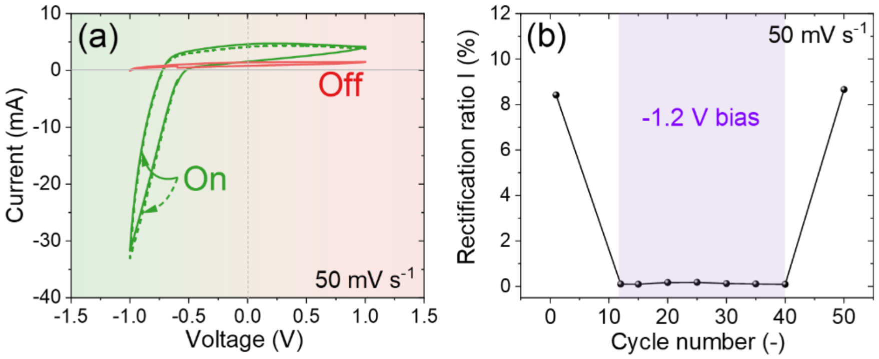

Fig. S30. (**a**) CV curves and (**b**) the calculated *RR* changes in the on- and off-state of (Ti |1 M $H_3PW_{12}O_4$ | $C_{1.5}$) CAPode.

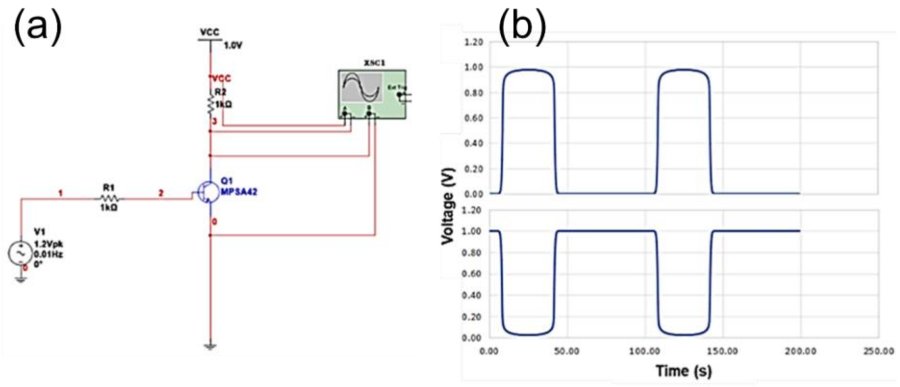

Fig. S31. (**a**) Scheme depicting for NOT logic circuit. (**b**) Input signals of (0), (1), and corresponding outputs.

To demonstrate the potential of capacitive computing with ion-selective redox ionologic devices, in the following, CAPode and G-CAPode are integrated to construct a logic NAND gate (**Fig. S32a**). A logic gate consisting of two CAPodes, one G-CAPode (coin cells setups), and two 1 kΩ resistors.

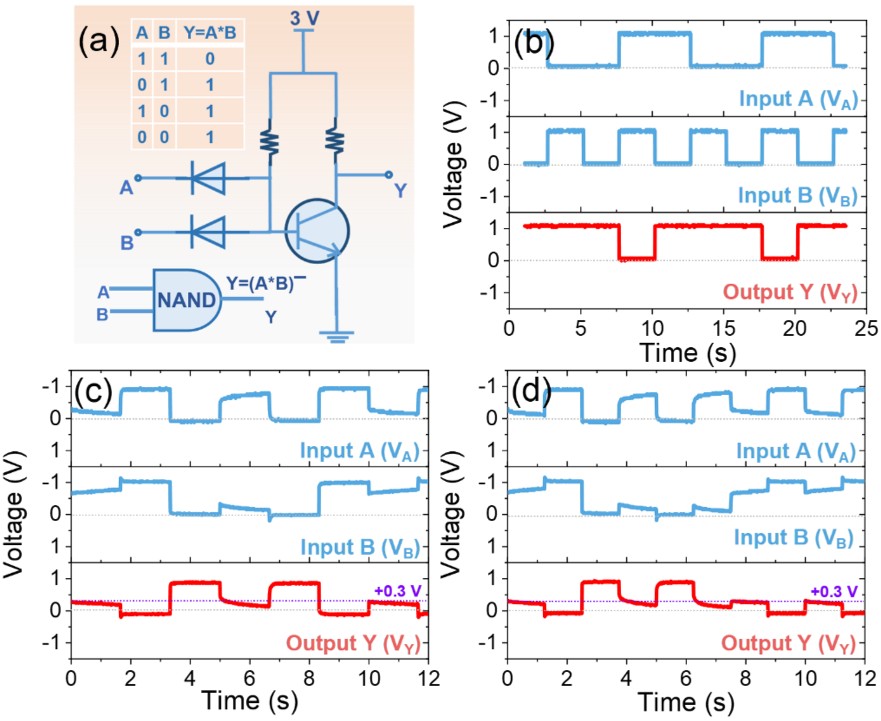

Fig. S32. (**a**) Scheme depicting for NAND logic circuit. (**b**) Input signals of (0,0), (1,0), (0,1), (1,1), and corresponding outputs at the frequency V_A/V_B = 0.1/0.2 Hz of commercial diodes and transistors for NAND logic circuits. Input signals of (0,0), (1,0), (0,1), (1,1), and corresponding outputs of as-designed CAPodes and G-CAPode for NAND logic circuits at the frequency V_A/V_B = (**c**) 0.3/0.1 Hz and (**d**) 0.4/0.1 Hz, respectively.

Input signals of (0,0), (1,0), (0,1), (1,1) and corresponding outputs at the frequency V_A/V_B = 0.1/0.2 Hz of commercial diodes and transistors for NAND logic circuits are shown in **Fig. S32b**. The results in **Fig. S32c** demonstrate that the output at the frequency V_A/V_B = 0.3/0.1 Hz is low ($V_Y \approx 0$ V) only when V_A and V_B are both high ("1" = -1 V), and the output is high ("1" = 0.3 V), as long as one of the inputs is low. These results correspond well to the logic function scheme of input signals of (0,0), (1,0), (0,1), (1,1) for commercial diodes and transistors in NAND circuit (**Fig. S32b**), although the output voltage of "1" (0.3 V) is significantly lower than the ideal 1 V probably due to the high resistance near the G-CAPode, which results in the high IR drop. Consequently, a simple NAND logic has been realized with the ionologic CAPode devices. When increasing the frequency (V_A/V_B = 0.4/0.1 Hz) (**Fig. S32d**), the output voltages are coincident with the output values when a lower frequency is applied (**Fig. S32c**). It should be also noted that the voltage output of (0,0) input is higher than outputs for

(0,1) and (1,0) inputs. For the NAND gate based on conventional diodes and transistors intrigued by electrons is extremely close to 0 V. This difference is due to the fact that the CAPodes and G-CAPodes are built by hybrid ECs, which also exhibit the characteristics of charging and discharging curves in ECs.

16. Comparing Systems

Table S3. The distinction between electrochemical capacitor diodes (CAPodes), ion transistors, and electrochemical capacitor transistors (G-CAPodes).

	CAPodes	Ion transistors	G-CAPodes	Ref.
**Definition**	an ionic diode with super-capacitance	electrolyte-gated transistors (including organic electrochemical transistor (OETC)), electrolyte-gated field-effect transistor (EGFET), redox-active ion transistors and so on), ionic FET, ionic bipolar junction transistors	as an ionic FET with super-capacitance	34-56
**Functionality**	diode	transistor	transistor	34-56
**Mechanism**	ion sieving/ redox reaction	ion doping (OETC) /ion depletion (**EGFET**) redox-gating	redox- potential controlling	34-56
**Electrode set-up**	porous carbons with precise pores distribution, redox or intercalation active materials	conducting polymers, conductive metals, semiconductors	porous carbon, redox or intercalation active materials	34-56
**Capacitance**	super high (~100 F g^{-1} at 2 mV s^{-1}), ref. 14	general moderate (10 to 20 μF/cm^2 in a typical metal–insulator–metal structure), ref. 29	super high (~93 F g^{-1} at 2 A g^{-1}), ref. 17 and 20	25, 40, 28 and 31
**Working voltage**	Low knee voltage (0.1 V), ref. 14	Moderate threshold voltage (-1.0 V), ref. 29	Low threshold voltage (0.2 V), ref. 20	25, 40, 31
**Energy consumption**	minimal, storing energy in a high-capacity material, ref. 14	very limited capacity for energy storage, ref. 29	minimal, storing energy in a high-capacity material, ref. 20	25, 40, 31
**Frequency-dependent impedance characteristics**	high impedance at a limited working frequency of 10 Hz	low impedance at high working frequency ~ 1000 Hz	high impedance at a limited working frequency of 10 Hz	25, 40, 31
**Applications**	logic operations, applications required frequency-dependent resistance, energy storage	logic operations and circuit switching	logic operations, and circuit switching applications required frequency-dependent resistance, energy storage	34-56

Table S4. Comparing differences from redox gating and organic electrochemical transistors and key prior works with this work.

	**CAPode**	**G-CAPode**	**Redox gating transistor**	**Organic electrochemical transistor**	**CAPode + G-CAPode**
Reference	35-39	40	41-43	44-48	this work
Mechanism	ion sieving/ redox reaction	redox-potential controlling	redox-potential controlling	electrochemical ion doping	asymmetric polarization for redox + redox-potential controlling
Functionality	diode	transistor	transistor	transistor	multifunctionality (diode and transistor)
Electrode Set-up	WE: carbon CE: carbon	WE: carbon CE: carbon GE: carbon	WE: metal oxides CE: carbon GE: carbon	organic semiconductors	WE: Ti, also as collector, eco-freindly) CE and GE: cheap and available porous carbon
Electrolyte Set-up	toxic organic solvent, acid or alkali	toxic acid	ionic liquids	ionic liquids are frequently used	non-toxic aqueous redox electrolyte with high electrochemical stability, eco-friendly solvents
Size of channel	micrometer	micrometer	micrometer	Possible to be 100 nm	micrometer
Capacitance	high (~2.52 mF cm^{-2} at 10 mV s^{-1})	high (6.9 mF cm^{-2} at 10 mV s^{-1})	low (10 to 20 μF cm^{-2} in a typical metal–insulator–metal structure)	general moderate (~40 F cm^{-3} in PEDOT:PSS system)	high (~14 mF cm^{-2} at 10 mV s^{-1})
Working voltage	Low knee voltage (~0.1 V)	Low threshold voltage (~−0.2 V)	high (1.4 – 1.5 V)	moderate threshold voltage (~–0.3 V at Polymer electrode and aqueous electrolyte system)	Low knee voltage (~0.1 V) Low threshold voltage (~−0.2 V)
Energy consumption	minimal, storing energy in a high-capacity material	minimal, storing energy in a high-capacity material	very limited capacity for energy storage	very limited capacity for energy storage	minimal, storing energy in a high-capacity material
Logic gate integration in one device	no	no	no	no	intergration of CAPode and G-CAPode
Suitable for bioelectronic applications	no	no	no	yes	yes

Overall, due to the specific polarization of the Ti and carbon electrode when the system is repolarized, the constructed CAPode can effectively store the charge as a result of a redox reaction only when the Ti electrode is negatively polarized. Compared to other similar published works (**Table S4**), our work shows different properties on the following

aspects, including mechanism, functionality, electrode set-up, electrolyte, size of channels, EDL capacitance, threshold voltage and energy consumption, rectification performance, and logic gate integration. Especially, the proposed CAPode and G-CAPode based on eco-friendly aqueous electrolytes and electrodes, enhanced ionic and electronic conductivity, stability in aqueous environments, eco-friendly processing, and versatile application potential in bioelectronics. These features make CAPode and G-CAPode highly suitable for advanced bioelectronic applications, providing reliable performance and compatibility with biological systems.

Supplementary References

1. Huang, H.-H., Chen, J.-Y., Lin, M.-C., Wang, Y.-T., Lee, T.-L. and Chen, L.-K. Blood responses to titanium surface with TiO_2 nano-mesh structure. *Clinical Oral Implants Research* **23**, (2012).
2. David, E. and Thomas, W. Titanium dioxide–electrolyte interface. Part 2.—Surface charge (titration) studies. *Journal of the Chemical Society, Faraday Transactions 1: Physical Chemistry in Condensed Phases* **76**, (1980).
3. Allodi, V., Brutti, S., Giarola, M., Sgambetterra, M., Navarra, M.A., Panero, S., Mariotto, G. Structural and Spectroscopic Characterization of A Nanosized Sulfated TiO_2 Filler and of Nanocomposite Nafion Membranes. *Polymers* **8**, （2016).
4. Zheng, T., Xiong, J., Shi, X., Zhu, B., Cheng, Y.-J., Zhao, H., Xia, Y. Cocktail therapy towards high temperature/high voltage lithium metal battery via solvation sheath structure tuning. *Energy Storage Materials* **38**, (2021).
5. Wang, Z. C., Sun, Y. Y., Mao, Y. Y., Zhang, F. R., Zheng, L., Fu, D. S., Shen, Y. B., Hu, J. C., Dong, H. L., Xu, J. J., Wu, X. D. Highly concentrated dual-anion electrolyte for non-flammable high-voltage Li-metal batteries. *Energy Storage Materials* **30**, (2020).
6. Wang, J., Polleux, J., Lim, J. & Dunn, B. Pseudocapacitive contributions to electrochemical energy storage in TiO_2 (anatase) nanoparticles. *Journal of Physical Chemistry C* **111**, (2007).
7. Augustyn, V., Simon, P. & Dunn, B. Pseudocapacitive oxide materials for high-rate electrochemical energy storage. *Energy and Environmental Science* **7**, (2014).
8. Ye, J., Tan, H., Wu, S., Ni, K., Pan, F., Liu, J., Tao, Z., Qu, Y., Ji, H., Simon, P., Zhu, Y. *Advanced Materials* **30**, (2018).
9. Funke, H.; Scheinost, A. C.; Chukalina, M. Wavelet analysis of extended X-ray absorption fine structure data. *Physical Review* **71**, (2005).
10. Zhu H., Tain R and Rhodes C. A study of the decomposition behaviour of 12-tungstophosphate heteropolyacid in solution. *Canadian Journal of Chemistry* **81**, (2003).
11. Skunik-Nuckowska, M. et al. Capacitance characteristics of carbon-based electrochemical capacitors exposed to heteropolytungstic acid electrolyte. *Electrochimica Acta* **282**, (2018).

12. Marcì, G. et al. Keggin heteropolyacid $H_3PW_{12}O_{40}$ supported on different oxides for catalytic and catalytic photo-assisted propene hydration. *Phys. Chem. Chem. Phys.* **15**, (2013).
13. Rafiee, E. & Eavani, S. $H_3PW_{12}O_{40}$ supported on silica-encapsulated γ-Fe_2O_3 nanoparticles: A novel magnetically-recoverable catalyst for three-component Mannich-type reactions in water. *Green Chemistry* **13**, (2011).
14. Iimura, R., Hasegawa, T. & Yin, S. Electrochromic Behavior Originating from the W6+/W5+Redox in Aurivillius-type Tungsten-Based Layered Perovskites. *Inorg. Chem.* **61**, (2022).
15. Li, Q. et al. Tunable and sustainable photocatalytic activity of photochromic Y-WO3under visible light irradiation. *RSC Adv.* **11**, (2020).
16. Li, X. et al. Toward a Practical Zn Powder Anode: $Ti_3C_2T_x$ MXene as a Lattice-Match Electrons/Ions Redistributor. *ACS Nano* **15**, (2021).
17. Petrov, M. M. et al. Electrochemically driven evolution of Br-containing aqueous solution composition. *J. Electroanal. Chem.* **836**, (2019).
18. Zhao, Y. et al. A reversible Br_2/Br^- redox couple in the aqueous phase as a high-performance catholyte for alkali-ion batteries. *Energy Environ. Sci.* **7**, (2014).
19. Nakka, L., Molinari, J. E. & Wachs, I. E. Surface and bulk aspects of mixed oxide catalytic nanoparticles: Oxidation and dehydration of CH3OH by polyoxometallates. *Journal of the American Chemical Society* **131**, (2009)
20. El Arrouji, I. *et al.* Nh3-selective catalytic reduction of nox to n2 over ceria supported wox based catalysts: Influence of tungsten content. *Catalysts* **11**, (2021).
21. Martinelli A., Matic A., Jacobsson P., Börjesson L., Navarra M.A., Munaò D., Panero S., Scrosati B. A study on the state of PWA in PVDF-based proton conducting membranes by Raman spectroscopy. *Solid State Ionics* ***178*** *(2006).*
22. Okuhara, T., Mizuno, N., Misono, M. Catalytic Chemistry of Heteropoly Compounds. *Advances in Catalysis* **41** (1996).
23. Itoh, T. & McCreery, R. L. In situ Raman spectroelectrochemistry of electron transfer between glassy carbon and a chemisorbed nitroazobenzene monolayer. *Journal of the American Chemical Society* **124**, (2002).
24. Anne Damian, H., POLYOXOMETALATES AND PEPTIDES: hybridisation and disulfide detection. *PhD thesis*, University of Nottingham, (2022).

25. Zhang, L. *et al.* Boosting Electroreduction Kinetics of Nitrogen to Ammonia via Atomically Dispersed Sn Protuberance. *Angewandte Chemie - International Edition* **62**, (2023).
26. Zabinsky, S. I., Rehr, J. J., Ankudinov, A., Albers, R. C. & Eller, M. J. Multiple-scattering calculations of x-ray-absorption spectra. *Physical Review B* **52**, (1995).
27. Ravel, B. & Newville, M. ATHENA, ARTEMIS, HEPHAESTUS: Data analysis for X-ray absorption spectroscopy using IFEFFIT. *Journal of Synchrotron Radiation*. **12**, (2005).
28. Ankudinov, A. & Ravel, B. Real-space multiple-scattering calculation and interpretation of x-ray-absorption near-edge structure. *Physical Review B* **58**, (1998).
29. Funke, H., Scheinost, A. C. & Chukalina, M. Wavelet analysis of extended x-ray absorption fine structure data. *Physical Review B* **71**, (2005).
30. Ravel and M. Newville, ATHENA, ARTEMIS, HEPHAESTUS: data analysis for X-ray absorption spectroscopy using IFEFFIT, *Journal of Synchrotron Radiation* **12**, (2005).
31. Zabinsky, S. I.; Rehr, J. J.; Ankudinov, A.; Albers, R. C.; Eller, M. J. Multiple-Scattering Calculations of X-Ray-Absorption Spectra. *Physical Review B* **52**, (1995).
32. Moya, A.A. Identification of characteristic time constants in the initial dynamic response of electric double layer capacitors from high-frequency electrochemical impedance. *Journal of Power Sources* **397**, (2018).
33. Nguyen, T.Q and Breitkopf, C. Determination of Diffusion Coefficients Using Impedance Spectroscopy Data. *Journal of The Electrochemical Society* **165**, (2018).
34. Zhang, E., Fulik, N., Hao, G.-P., Zhang, H.-Y., Kaneko, K., Borchardt, L., Brunner, E., Kaskel, S. An Asymmetric Supercapacitor–Diode (CAPode) for Unidirectional Energy Storage. *Angewandte Chemie International Edition* **58**, (2019).
35. Zhou, H. et al. General Design Concepts for CAPodes as Ionologic Devices. *Angewandte Chemie - International Edition* **62**, (2023).
36. Gellrich, C., Shupletsov, L., Galek, P., Bahrawy, A., Grothe, J., Kaskel, S. A Precursor-Derived Ultramicroporous Carbon for Printing Iontronic Logic Gates and Super-Varactors. *Advanced Materials* **36**, (2024).

37. Tang, P., Tan, W., Li, F., Xue, S., Ma, Y., Jing, P., Liu, Y., Zhu, J., Yan, X. A Pseudocapacitor Diode Based on Ion-Selective Surface Redox Effect. Adv. Mater. *Advanced Materials* **35**, (2023).
38. Bahrawy, A., Galek, P., Gellrich, C., Grothe, J., Kaskel, S. Advanced Redox Electrochemical Capacitor Diode (CAPode) Based on Parkerite (Ni3Bi2S2) with High Rectification Ratio for Iontronic Applications. *Advanced Functional Materials* **34**, (2024).
39. Ma, H., Liang, J., Qiu, J., Jiang, L., Ma, L., Sheng, H., Shao, M., Wang, Q., Li, F., Fu, Y., Wang, J., Xie, E., Chai, Y. and Lan, W. A Biocompatible Supercapacitor Diode with Enhanced Rectification Capability toward Ion/Electron-Coupling Logic Operations. *Advanced Materials* **35**, (2023).
40. Lochmann, S., Bräuniger., Gottsmann, Y. V., Galle, L., Grothe, J., Kaskel, S. Switchable Supercapacitors with Transistor-Like Gating Characteristics (G-Cap). *Advanced Functional Materials* **30**, (2020).
41. Bahrawy, A., Galek, P., Gellrich, C., Niese, N., Grothe, J., Kaskel, Stefan. A gated highly variable pseudocapacitor based on redox-window control (G-CAPode). *Energy Storage Materials* **74**, (2025).
42. Zhang, L., Liu, C., Cao, H., Erwin, A. J., Fong, D. D., Bhattacharya, A., Yu, L., Stan, L., Zou, C., Tirrell, M. V., Zhou, H., Chen, W. Redox Gating for Colossal Carrier Modulation and Unique Phase Control. *Advanced Materials* **35**, (2024).
43. Cao, H., Liu, C.J., Fong, D. D., Bhattacharya, A., Tirrell, M. V., Zhou, H., Chen, W. Redox gating-induced modulation of charge carrier density and lattice expansion in LaNiO3 thin films. *Applied Physics Letters* **125**, (2024).
44. Raza, A., Farooq, U., Naseem, K., Alam, S., Khan, M. E., Mohammad, A., Zakri, W., Khan, M. Y. A focused review on organic electrochemical transistors: A potential futuristic technological application in microelectronics. M*icrochemical Journal* **207**, (2024).
45. Rivnay, J., Inal, S., Salleo, A. et al. Organic electrochemical transistors. *Nature Reviews Materials* **3**, (2018).
46. Guo, J., Chen, S.E., Giridharagopal, R. et al. Understanding asymmetric switching times in accumulation mode organic electrochemical transistors. *Nature Materials* **23**, (2024).
47. Huang, W., Chen, J., Yao, Y. et al. Vertical organic electrochemical transistors for complementary circuits. *Nature* **613**, (2023).

48. Mei, T., Liu, W., Xu, G., Chen, Y., Wu, M., Wang, L., Xiao, K. Ionic Transistors. *ACS Nano* **18**, (2024).
49. Zhang, H., Yu, J., Yang, X., Gao, G., Qin, S., Sun, J., Ding, M., Jia, C., Sun, Q and Wang, Z. Lin. Ion Gel Capacitively Coupled Tribotronic Gating for Multiparameter Distance Sensing. *ACS Nano* **14**, (2020).
50. Zhang, B., Zheng, Y., Chen, W. et al. Differential capacitance in ion-gel-gated organic transistors investigated by impedance spectroscopy. *Ionics* **24**, (2018).
51. Sayago, J., Soavi, F., Sivalingam, Y., Cicoirac, F and Santato, C. Low voltage electrolyte-gated organic transistors making use of high surface area activated carbon gate electrodes. *Journal of Materials Chemistry C* **2**, (2014).
52. Yeh, P. C., Lin, Y. W et. al. Threshold voltage controlled by gate area and gate recess in inverted trapezoidal trigate AlGaN/GaN MOS high-electron-mobility transistors with photoenhanced chemical and plasma-enhanced atomic layer deposition oxides. *Applied Physics Express* **8**, (2015).
53. Zhong D., Zhao, C., Liu, L., Zhang, Z., Peng, L. M. Continuous adjustment of threshold voltage in carbon nanotube field-effect transistors through gate engineering. *Applied Physics Letters* **112**, (2018).
54. Carneiro-Neto, E. B., Li, Z., Pereira, E., Mathwig, K., Fletcher, P. J., Marken, F. Understanding Transient Ionic Diode Currents and Impedance Responses for Aquivion-Coated Microholes. *ACS Applied Materials Interfaces* **33**, (2023).
55. Abouelamaiem, D. I., He, G., Neville, T. P., Patel, D., Ji, S., Wang, R., Parkin, I. P., Jorge, A., Titirici, B. M-M., Shearing, P. R., Brett, D. J.L. Correlating electrochemical impedance with hierarchical structure for porous carbon-based supercapacitors using a truncated transmission line model. *Electrochimica Acta* **284**, (2018).
56. Liu, F., Xie, W., Shi, S., Frisbie, C. D., Ruden, P. P. Coupling of channel conductance and gate-to-channel capacitance in electric double layer transistors. *Applied Physics Letters* **103**, (2013).
